# Applications and Outcomes of Internet of Things for Patients with Alzheimer's Disease/Dementia: A Scoping Review

**DOI:** 10.1155/2022/6274185

**Published:** 2022-03-15

**Authors:** Abbas Sheikhtaheri, Farveh Sabermahani

**Affiliations:** Department of Health Information Management, School of Health Management and Information Sciences, Iran University of Medical Sciences, Tehran, Iran

## Abstract

**Objectives:**

We aimed to identify and classify the Internet of Things (IoT) technologies used for Alzheimer's disease (AD)/dementia as well as the healthcare aspects addressed by these technologies and the outcomes of the IoT interventions. *Methodology*. We searched PubMed/MEDLINE, IEEE Explore, Web of Science, OVID, Scopus, Embase, Cochrane, and Google Scholar. In total, 13,005 papers were reviewed, 36 of which were finally selected. All the reviews were independently carried out by two researchers. In the case of any disagreement, the problem was resolved by holding a meeting and exchanging views. Due to the diversity of the reviewed studies, narrative analysis was performed.

**Results:**

Among the technologies used for the patients including radio frequency identification (RFID), near field communication (NFC), ZigBee, Bluetooth, global positioning system (GPS), sensors, and cameras, the sensors were employed in 36 studies, most of which were switch and vital sign monitoring sensors. The most common aspects of AD/dementia care monitored using these technologies were activities of daily living (ADLs) in 27 studies, followed by sleep patterns and disease diagnosis in 19 and 14 studies, respectively. Sleeping, medication, vital signs, agitation, memory, social interaction, apathy, movement, tracking, and fall were other aspects monitored by IoT. Then, their outcomes were reported.

**Conclusion:**

Using IoT for AD/dementia provides many opportunities for considering various aspects of this disease. Moreover, the ability to use various technologies for gathering patient-related data provides a comprehensive application for almost all aspects of the patients' care with high accuracy.

## 1. Introduction

Alzheimer's disease (AD) is a chronic brain disease that worsens over time and may result in dementia. It begins with a few brain changes. People with AD/dementia experience significant symptoms over time, such as language impairment and memory loss. The nerve cells in the regions of the brain that allow a person to execute fundamental actions like walking and eating are impaired as the disease progresses and become ultimately fatal [1, 2]. Around 6.2 million Americans aged 65 and older have AD/dementia, and this number is expected to rise to 13.8 million by 2060. AD/dementia is the sixth leading cause of mortality in the United States and the fifth leading cause of death among people aged 65 and higher. AD/dementia mortality increased by more than 145% in 2000-2019. However, deaths from heart disease, as the first leading cause of mortality, decreased by 7.3% [3, 4].

Patients with AD/dementia face various problems, including sleep disturbance, inability to perform daily activities properly, lack of discipline in taking medication, agitation, forgetfulness, isolation, and apathy [5–8]. In 2020, about 11 million family members and other unpaid caregivers supplied around 15.3 billion hours of care to people with AD/dementia [9].

Caregiving is referred to as taking care of another person's health and well-being [10, 11]. Caregivers provide emotional support to people with AD/dementia, establish communication and coordinate care with other family members and healthcare providers, ensure safety at home and other places, and manage health conditions [9]. Given that providing healthcare for AD/dementia patients become challenging as the society ages, these patients' caring needs could be somehow met using Internet of Things- (IoT-) based technologies [12, 13].

In the IoT, all the objects can be connected to anyone and anything at any time and any place on any service or network with only a unique Internet address [14]. Healthcare is among the important applications of IoT, so that the impact of IoT innovations is noticeable in this field. Using IoT data gathering capabilities, this technology can be integrated with other health information and clinical technologies [15]. IoT enables continuous and immediate communication with patients, provides healthcare by gathering accurate data, and significantly reduces costs and waiting time for patients [16, 17].

Several studies have used IoT-based technologies to respond to different supportive needs of AD/dementia patients. A large number of studies have investigated IoT for AD/dementia patients so far due to different stages of AD (mild cognitive impairment (MCI), Alzheimer's and dementia) [18] as well as the wide range of healthcare aspects and needs of patients with AD/dementia [6, 19]. Some studies, for example, have examined sleep patterns, daily activities, agitation, and tracking AD/dementia patients using IoT-based systems [20–24]. Moreover, the scope of IoT innovations and research includes a variety of data gathering technologies, each of which has a number of advantages and disadvantages. It is very important to assess what aspects of AD/dementia patients' care each technology can target [25]. Technologies such as radio frequency identification (RFID), near field communication (NFC), cameras, and sensors have been employed in various studies to support these patients and have alleviated their problems to some extent [26–29].

Previous reviews on IoT technology are not focused on AD/dementia and have addressed neurodegenerative diseases [30–36]. One study evaluated technology-based outcome measures related to neurodegenerative disorders in clinical trials [32]. Some other reviews have examined only one specific dimension of AD/dementia such as physical activity monitoring [7, 37]. Other studies have investigated the possibility of disease diagnosis using this technology [25, 37–39]. Some other works have only examined AD/dementia patients' wandering symptom using IoT [40, 41] or only those patients staying at home or nursing homes [39, 42, 43]. The present study is aimed at reviewing the scope of different IoT applications and technologies for AD/dementia patients, along with their outcomes.

### 1.1. IoT Applications and Limitations in Healthcare

IoT is a global network of interconnected things that uses the Internet for their connection. The IoT allows connections to be made between physical things and people or even between objects, transforming the actual world into a digital one. Furthermore, IoT devices previously connected to a local network can now connect to broader networks through the Internet. Using communication technology and sensors, common things such as clothes, books, and furniture can connect with more complicated devices such as computers and smartphones in the IoT technology. Continuous or intermittent data streams can be collected, preprocessed, and analyzed by sensors [44, 45]. Using sensors and launching IoT-based systems are not particularly expensive; the usage of these systems is cost-effective owing to their functionality and efficiency, especially due to the diversity and developments in sensor manufacturing and lower production costs [45, 46].

IoT is employed in a variety of industries, such as healthcare. The goal of implementing IoT in many businesses is to link and coordinate conventional industries over the Internet in order to provide more efficient services. One of the main aims of IoT is to improve people's quality of life. In reality, IoT has a significant impact on human behaviors. It may be utilized in a variety of ways, such as creating smart homes or putting sensors in wearables. IoT applications involve improving data gathering methods and overcoming problems with earlier data collection methods because of conditions such as faraway data collection [46].

In the field of healthcare, IoT offers several applicability. Measures of health and behavior can be tracked by collecting and evaluating health-related data and the individuals' personal living environments. Remote monitoring and emergency notification systems employ the IoT, and customized sensors can be used to monitor health of the elderly, pregnant women, persons with chronic conditions, and a variety of other people. Furthermore, IoT may be utilized to detect and control diseases as well as track medicine usage [21, 47, 48].

In an emergency, IoT-based detection and monitoring devices can be used to gather and store health data and save a patient's life, especially for those with specific disorders (such as cancer, diabetes, and Alzheimer's). Complex medical technologies, such as artificial hearts, joints, and organ transplants, may also be able to interact with patients on their own [21, 44, 49, 50]. Using the IoT provides many benefits in areas such as patient health, safety, and security of pharmaceutical products by monitoring their production processes, attaching smart labels to drugs, and tracking their supply chain [44, 49, 50].

It is feasible to make suitable decisions for each patient utilizing data acquired from medical IoT devices attached to patients and advanced algorithms to evaluate the data. Temperature sensors can detect patient's body temperature, while also modern medical equipment and home robots are other examples of IoT-based health monitoring systems. Another use of IoT is the usage of smart beds connected to the Internet for determining the precise time of the patient's presence in bed [44]. In the health domain, the requirement for real-time or near-real-time data access is critical, and IoT can provide access to such data [49, 50].

Collecting patient's vital signs using a network of sensors connected to medical equipment and patient's body as well as sending the data to the cloud platform of an appropriate medical center (for example, a hospital) for storage and processing is other examples of services that can be provided in the field of health using IoT [21, 50].

Fall detection, monitoring of physical activity of the elderly, medical refrigerators, athlete care, patient monitoring, management of chronic diseases, reduction of ultraviolet rays- (UV-) related injuries, pollution control (hand hygiene control), sleep control, and dental health are some of the main application areas of IoT in the health sector [21, 44, 45, 47, 48].

**Figure 1:**
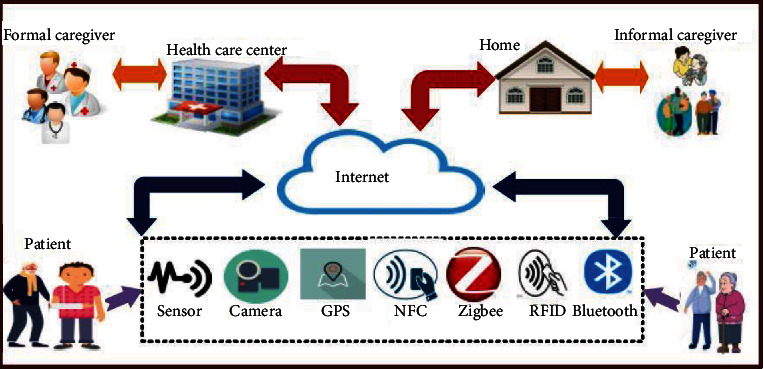
IoT applications in healthcare.

There are several advantages to prevention, monitoring, and telemedicine in this field. This technology also allows for more comprehensive and less expensive medical services for patients [21, 47–50]. IoT applications in the health field are demonstrated in Figure 1.

IoT, similar to any other technologies, has its own constraints. In some instances, the efficiency of sensor data processing, power consumption, and battery power of sensors and other devices in IoT systems may limit its utilization. In some contexts, the difficulty of implementing and deploying an IoT system might be a restriction for IoT. Another limitation of IoT may be what is known as compliance. IoT requires its own set of laws and regulations. However, the IoT rules have not yet been extensively applied, and the existing guidelines have in some cases made IoT-based systems more complex. Large amounts of data are among the obstacles of its utilization, and the essential procedures to store and retrieve the data must be addressed [21, 44, 45, 47]. Lack of trust in security and privacy, unpredictability of system performance and service quality, legal issues related to data ownership, and poor acceptability and acceptance of IoT systems by patients and healthcare providers are all IoT challenges in the field of health that need to be addressed [21, 47–50].

## 2. Material and Methods

This study is a scoping review [51]. In this type of review, all the relevant studies are considered, but their quality is not usually evaluated. In the present study, the scope of the studies conducted on using IoT for the care of AD/dementia patients was reviewed. We applied the preferred reporting items for systematic reviews and meta-analysis extension for scoping review (PRISMA-ScR) criteria (supplementary data 1, Table S1). In all the stages, the number of studies and reasons for the exclusion was reported according to PRISMA-ScR criteria [52].

### 2.1. Data Sources and Search Strategy

We searched PubMed/MEDLINE, IEEE Explore, Web of Science (WoS), OVID, Scopus, Embase, Cochrane, and Google Scholar. The keywords were selected using MeSH and EMtree terms as well as their synonyms. Moreover, some keywords were added during the initial review of the related papers. Searching in the databases was carried out by combining keywords such as Alzheimer's disease, dementia, IoT, RFID, patient monitoring, assistive technology, wireless sensor network, ZigBee, Bluetooth, NFC, ubiquitous computing, global positioning system (GPS), global system for mobile communications (GSM), general packet radio services (GPRS), smart environments, machine-to-machine communications, and physical activity monitoring. The full search strategy in each of the databases is listed separately in Table S1 included in the supplementary data 2.

In this study, the search was performed based on the population, intervention, control, and outcome (PICO) criteria as follows:

*Population*: patients at different stages of AD and dementia were included.

*Intervention*: any type of IoT intervention such as RFID, sensors, cameras, and NFC was included.

*Control*: all the studies with or without control groups were reviewed.

*Outcome*: any outcomes reported in the papers were reviewed, such as activity of daily living (ADL), taking medication, tracking, and locating the physical position of the elderly.

### 2.2. Inclusion and Exclusion Criteria

We did not limit our search to any languages. Following the introduction of the IoT in 1999, we considered all the papers published from the beginning of 1997 to the beginning of 2021.  Table 1 presents the inclusion and exclusion criteria.

After removing the duplicates, we reviewed the titles of the studies; if they were relevant to our objectives, their abstracts were also reviewed. We then reviewed the full text of the relevant abstracts. If the study met our objectives, they entered the final review. Moreover, references of the selected studies were reviewed to retrieve the related papers. In all the stages, two independent researchers reviewed the articles. In the case of any disagreement, the problem was resolved by holding a meeting and exchanging the views.

### 2.3. Data Extraction and Analysis

We developed a data extraction form to extract data from the studies, which included information about the author's name, date of publication, country of the study, problems of AD/dementia patients tracked using IoT technology, type of the proposed and used IoT technologies to solving these problems, IoT intervention description, intervention location, intervention duration, and characteristics of the participants. Also, information about the results of using these technologies, such as disease diagnosis, medication management, and proper and independent performance of ADLs, was extracted. In all the stages, data were extracted by two independent researchers, and in the case of any disagreement, the problem was resolved by exchanging views in regular meetings.

After extracting the data, the problems of AD/dementia patients and type of technologies as well as results and outcomes of using these technologies were classified by narrative synthesis.

## 3. Results

### 3.1. Included Studies

**Figure 2:**
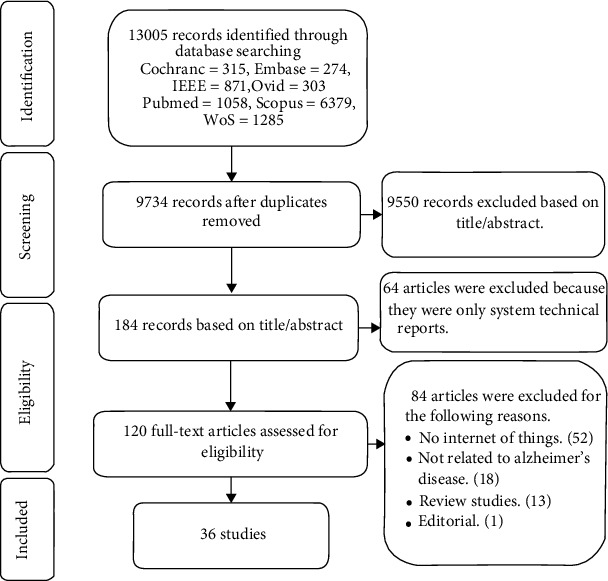
PRISMA flow diagram of study identification.

As shown in Figure 2, 36 out of 13,005 initial papers were finally selected. Because the IoT can contain a wide range of technologies, the researchers developed their search strategy to cover all these IoT technologies. However, terms similar to technology (nanotechnology, ultrasound technology, image technology, etc.), sensor (sensory, biosensor, immunosensor), GPS (similar to GPs standing for general physicians), and human area network (HAN; Han in China) were retrieved. Therefore, a large number of papers were retrieved; however, they were eliminated during the title/abstract screening step. Details of the final included studies in terms of interventions and outcomes are reported in Table S2 and Table S3 included in the supplementary data 2.

### 3.2. Geographical Distribution of Studies

**Figure 3:**
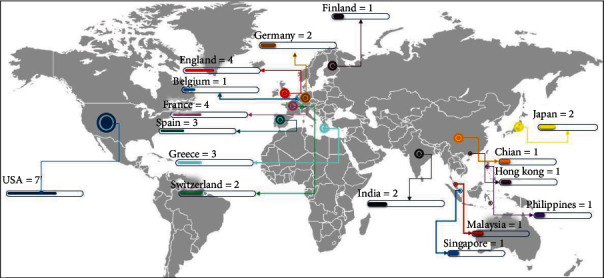
Geographical distribution of studies.

As shown in Figure 3, the United States had the highest amount of research (7 studies), followed by the United Kingdom and France (4 studies). Studies were done in seven Asian nations in addition to eight European countries, with Japan and India at the top of the list, with two studies each.

### 3.3. Distribution of Studies by Publication Year

**Figure 4:**
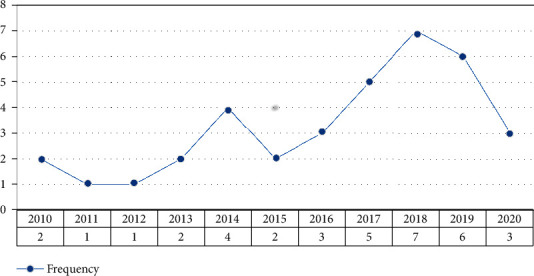
Distribution of the studies in terms of publication year.

Distribution of the articles by year of publication is depicted in Figure 4. The majority of papers in this topic were published in 2018. It is worth noting that the graph demonstrates general growth in IoT research for AD/dementia care over time, particularly during 2012-2014 and 2015-2018.

### 3.4. Intervention Duration and Place

**Figure 5:**
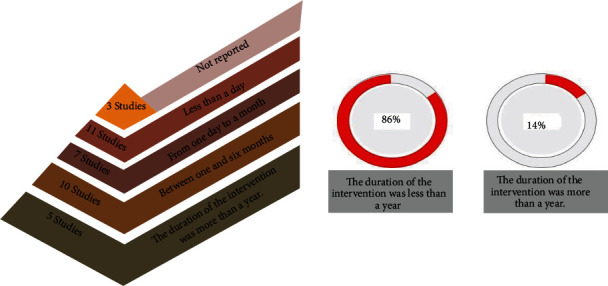
Intervention duration in different studies.

Because the type of technology, location, and duration of IoT interventions may all influence the outcomes of usage and satisfaction, the distribution of studies based on these factors is provided in this section. Length of the interventions in the trials included in this study varied widely. In 86% of the studies, the intervention duration was less than one year, and the rest of the studies had the intervention duration of more than one year. Figure 4 indicates the intervention duration in different studies. Furthermore, as shown in Figure 5, eleven studies had the intervention length of less than one day and only tested a system, not the long-term effect of the technology.

**Figure 6:**
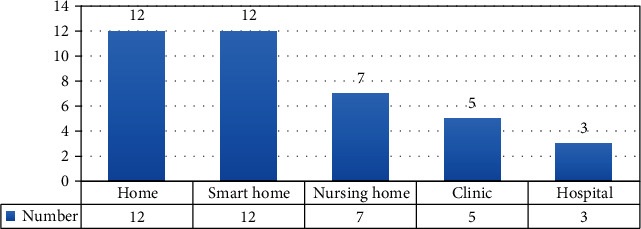
Intervention place in different studies.

Given that one of the purposes of using IoT for the persons with AD/dementia is to monitor their everyday life, results of an intervention will be more useful in practice if the intervention is implemented closer to the patient's usual living situations. Twelve of the included studies were in the patient's home, while the other 12 studies introduced interventions at smart homes that were set up for the research purposes. Nursing homes were the intervention sites in seven of the studies. Intervention and evaluation were carried out at the hospital in three studies.  Figure 6 displays the intervention locations. In several studies, the intervention was implemented in multiple locations.

### 3.5. Technologies Used for AD/Dementia Patients

The technologies used in the initial studies along with the general specifications of the studies are listed in Table S2 in the supplementary data 2.  Table 2 presents the technologies used in the studies along with their applications and limitations. Initial studies used RFID, NFC, ZigBee, and Bluetooth. In some works, GPS technology and different types of cameras were also employed. In addition to the mentioned technologies, sensors were used in all the studies.

### 3.6. Aspects of AD/Dementia Care Supported by IoT Technologies and Outcomes

Various studies have focused on diagnosing AD/dementia (14 studies) and monitoring a person's ADLs (27 studies), sleep patterns (19 studies), medication intake (9 studies), and vital sign monitoring (11 studies). Medication intake has been monitored to diagnose AD or provide better care for AD/dementia patients. Patient agitation (9 studies), monitoring memory status (5 studies), examining social interactions (4 studies), and apathy (3 studies) were other objectives of monitoring in the included studies. Movement (23 studies), tracking (12 studies), and fall (8 studies) were also some monitoring purposes.  Table 3 presents the objectives of monitoring and the obtained outcomes. Details of the outcomes and results of the interventions are reported in Table S3 in the supplementary data 2.

### 3.7. Technology Acceptance Evaluation

The technologies employed in the primary studies were also evaluated in terms of their acceptance and usability. These evaluations included feasibility (3 studies), usability (3 studies), and acceptability (3 studies). The feasibility was assessed for ADL, sleeping, movement, and tracking. Acceptability was also evaluated for ADL, sleeping, agitation, memory, movement, tracking, fall, and vital signs, whereas usability was evaluated for ADL, sleeping, movement, tracking, and diagnosis.  Table 4 summarizes the positive and negative feedbacks received on this issue.

## 4. Discussion

This study reviewed IoT technologies used for AD/dementia patients and their outcomes. Different types of sensors, cameras, GPS, Bluetooth, ZigBee, NFC, and RFID are among the technologies applied to detect objects and activities in support of AD/dementia patient care. Moreover, various dimensions of AD/dementia care are supported using IoT. Diagnosis, sleep, medication management, ADLs, vital signs, agitation, memory, social interactions, apathy, movement symptoms, tracking, locating, and risk of falling are among the care aspects supported by IoT with positive outcomes.

### 4.1. Diagnosis

The necessity to employ innovative technologies for the early detection of AD/dementia is not concealed for researchers due to the challenges with traditional AD/dementia diagnosis and the wide spectrum of this disease from mild cognitive impairment to dementia. Many studies have focused on using IoT technologies for the early diagnosis of AD/dementia (Table 3). These studies have diagnosed the disease at its early stages by monitoring person's behaviors and comparing them with those of people with AD/dementia [56, 59, 73, 74]. For example, in the proposed system for diagnosing MCI via monitoring the person's behaviors, the areas under the receiver operating characteristic (AUC) and precision-recall curves were obtained as 0.716 and 0.706, respectively [74]. For example, findings of the study on monitoring participant's activities indicated that the disease can be diagnosed via different activities with sensitivities for cooking (84.29%), eating (87.78%), toileting (94.79%), and getting ready for bed (92.38%) [57]. Findings of applying the random forest (RF), support vector machine (SVM), AdaBoost, and multilayer perceptron (MLP) algorithms on the data received from an IoT system for disease detection revealed that all these algorithms can generate substantial prediction models for AD/dementia diagnosis. Researchers, for example, were able to identify this condition with the best accuracy using RF (precision-recall curve = 0.73, *F*‐score = 0.77, sensitivity = 0.92) [73].

### 4.2. Sleeping

Sleep monitoring is performed using a variety of sensors and cameras as well as a combination of technologies. The data obtained from sleep monitoring have multiple applications in both diagnosing and caring for AD/dementia patients such as assessing sleep quality and quantity [26, 28, 58, 72], recognizing wandering during the night [27, 61, 62], diagnosing urinary tract infection [68], and finding the cause of cognitive decline during the day [22, 47]; for example, a system called Umemory was applied to examine the sleep quality of people with AD living at nursing homes. Sleep patterns were obtained from the sleep data. The sleep quality was evaluated by this system, and the average sleep efficiency was obtained as 32.1-80.0% [58]. Furthermore, using monitoring systems can improve sleep quality [67]. This positive effect can be due to identifying unusual cases through the system as well as using the relevant data to perform appropriate therapeutic interventions and improve cognitive status and other conditions such as patient's sleep quality [21, 49, 72].

The results of studies have indicated when a combination of several technologies (e.g., a combination of sensors and camera) is used for sleep monitoring; variables such as bed occupancy duration, maximum sleep time without moving, getting out of bed, frequency of changing position during sleep in an hour, and number of minutes with high and low movement are examined more accurately [21, 72]. Results of an IoT intervention revealed that total awake time spent in the bed for a night sleep decreased significantly from 3.79% to 22.1% of sleep time; this indicator decreased for all the participants. In addition, all the participants' total light sleep time decreased from 74.5% to 58.2% of total night sleep duration. The percentage of total nightly deep sleep time increased from 7.7% to 38.5%, and the frequency of overnight breaks was reduced in all the participants using this IoT system [21].

Regarding the accurate function of sleep monitoring systems, comparing sleep analysis obtained from automatic data with self-reported data of sleep and awake hours for diagnosing sleep/awake status indicated that accuracy, specificity, and sensitivity were obtained as 85%, 88%, and 73%, respectively [68]. The correlation between light sleep duration and cognitive tests was 0.836, while the correlation between total sleep duration and cognitive tests was 0.843, according to participants' sleep in another IoT-based intervention. As a result, monitoring sleep length and implementing IoT interventions to enhance sleep quality can assist patients in improving their cognitive state [47].

### 4.3. ADL

Monitoring daily activities has been extensively used for both diagnosing and taking care of AD/dementia patients. Most of the studies (75%) included in this review have monitored ADLs. Dimensions such as agitation [20–22, 61, 62], memory impairment [47, 49, 60, 73], social interactions [21, 26, 63, 67], and apathy [20, 59, 62] were all diagnosed based on the data obtained from monitoring the person's ADLs. Using reminders to perform ADLs could help the person live independently [48, 60].

An ADL monitoring through an IoT-based system revealed that when comparing the performance of the healthy and patient groups in the details of performed activities, the difference in time spent performing these activities was significant (*p* = 0.002). Furthermore, the total score between the two groups was significantly different (*p* > 0.0001) based on the scores obtained by performing the indicated activities. Making a phone call (*p* = 0.002), operating the television (*p* = 0.032), and retrieving objects (*p* ≤ 0.0001) were all significantly different between the groups. Furthermore, the findings of the study revealed 0.85 correlation between the overall score acquired from these activities and the mini mental state examination (MMSE) test, as well as -0.64 correlation between the time spent on these activities and the MMSE score. As a result, it is expected that by tracking everyday activities using an IoT-based system, one could gain a better understanding of a person's cognitive status [71].

### 4.4. Medication

Due to memory impairment in AD/dementia, a number of studies have focused on monitoring medication use by IoT [20, 21, 47, 63]. Discipline in medication use was considered as a factor for the early diagnosis of AD [20, 29, 63]. Monitoring the regular use of medication and performing interventions had a significant effect on AD/dementia patients' physical and cognitive health, which were accomplished by a variety of sensors, cameras, and technologies such as NFC [29, 47, 53, 60, 67]. The proposed systems in this field had positive effects on the patient's health status and methods such as sending reminders or alerts to patients or caregivers improved patients' medication use discipline and independent living [47, 53, 60]. One study, for instance, examined whether a person's medication indiscipline could be an early sign of AD/dementia. An electronic medicine box was used to record when the box was opened during the day. People with higher medication indiscipline were significantly more likely to be in the group with lower cognitive status [73].

### 4.5. Movement

Walking manner was considered an effective indicator for AD diagnosis [63, 65, 73]. The severity of walking problems among AD/dementia patients indicated their disease severity [53, 65]. Body positions such as sleeping, sitting, walking, and standing were monitored by inertial sensors and cameras, and the person's movement patterns were extracted [24, 60]; for example, the mean accuracy of recognizing sitting, sleeping, or standing positions was obtained as 97% and 99.1% by Naive Bayes (NB) and SVM algorithms, respectively [60]. A comparison was made between the movement patterns of healthy people and patients as well as the results of neuropsychological tests and movement patterns to validate the movement monitoring system [21, 47].

### 4.6. Fall

Falling was another movement hazard threatening the elderly, especially a person with cognitive impairment. Using pressure sensors and cameras to detect falls had acceptable results in preliminary studies [21, 60, 68, 72]. In one study, the proposed system could reduce the likelihood of falling in the intervention group compared to the control group [72].

### 4.7. Agitation

Agitation, which was among the prevalent problems among AD/dementia patients, could lead to secondary risks such as getting lost or going to dangerous places [76]. These incidents may occur in various environments, for each of which indoor- or outdoor-specific technologies were used (Table 2). Using reminder and alert systems had a great impact on controlling patients' wandering. The reminder system is an assistive technology for people with AD/dementia. This system basically provides information reminding a person of their daily tasks [27]. However, the alert system communicates with and alerts healthcare providers using computers, pagers, cell phones, and e-mail messages [77], an example of which is Escort system [27]. The results revealed that the patients' activities could be monitored, and their wandering could be improved using IoT-equipped robots. Robots can successfully perform activities such as returning a wandering patient to bed as well as recognizing and playing their favorite music at pleasant times [22].

### 4.8. Tracking

GPS patient tracking was among the most prevalent and cheapest methods used in the studies to diagnose agitation and wandering. By embedding GPS in small devices that had other features such as sending messages to patient's caregivers, these systems have provided promising results in preliminary studies [24, 48, 50]. However, GPS has some limitations: it has low accuracy indoors and positioning error of about 10 m outdoors [23]. Therefore, the studies affected by these limitations have used other methods, either independently or in combination with GPS, to track patients; for example, some studies have employed cameras, infrared sensors, Bluetooth, and ZigBee [21, 48, 59, 62].

The wireless transmitter range in an investigation was 51.97 m, thanks to the usage of an IoT-based system utilizing ZigBee and GPS. Depending on whether a patient is inside or outside the designated range, it sends appropriate messages. More than 82% of the announcements in this investigation were connected to the presence of patients at the distance of 48-55 m. The average reaction time to identifying the patient until the notification was 1.97 sec (standard deviation (SD) = 1.16) in this system. As a result, this system can be employed in situations when a patient's living environment has dimensions in this range, and it will be notified in less than 2 sec if a person is in a risky position [61].

**Figure 7:**
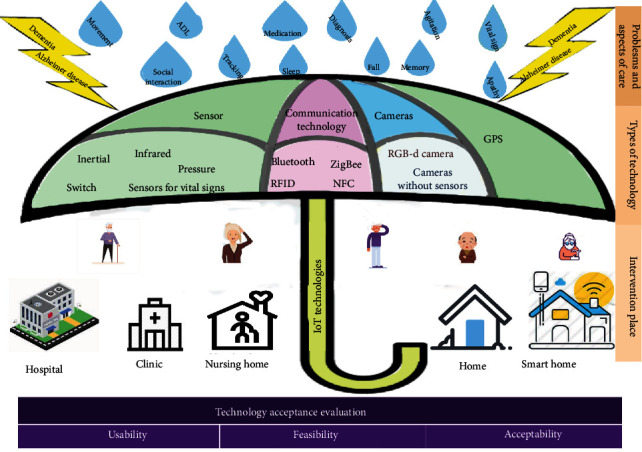
IoT technologies implemented and evaluated for various aspects of AD/dementia.

In summary, Figure 7 illustrates the IoT technologies, as well as aspects of AD/dementia that can be addressed with these technologies and the types of evaluations conducted in the included studies.

## 5. Limitations

Because our goal was to consider a wide range of IoT technologies and the outcomes of deploying these technologies, the study was not limited to a specific type of design, and the quality of the studies was not assessed.

## 6. Implications

Due to the rapid development of IoT systems and acceptance of this infrastructure by different communities, the results could serve as a guide for technology selection and development. The usage of a range of IoT data gathering technologies can assist diagnosis and care for AD/dementia. Meanwhile, combining a range of cameras with various sensors and presence detection technologies such as RFID, NFC, and Bluetooth can be beneficial in increasing the effectiveness of monitoring systems by overcoming the limits of each technology. Implications of employing these technologies include a wide variety of areas of patients' life, including all cognitive problems and everyday activities as well as sleep quantity and quality. Any individual with AD/dementia can receive a personalized care plan based on their lifestyle and health status in this way.

It is also crucial to choose the type of technology that better represents patients' requirements. A person with memory impairment, for example, may not have suitable selection of monitoring gadgets that should charge their batteries. In the case of agitated patients and number of times they are disturbed, it is preferable to avoid utilizing technologies in the form of wristbands or other wearable technologies that come into contact with the skin inconveniently.

Furthermore, researchers and joint technical and clinical groups deciding to set up an appropriate IoT-based system for AD/dementia patients could benefit from the results of this review. Researchers should conduct extra investigations and assessments for medication, apathy, and social interaction due to lack of information concerning feasibility, usability, and acceptability evaluations.

## 7. Conclusion

Due to the rapid advancement and reduction in the cost and dimensions of digital technologies, using IoT-based data gathering technologies can help diagnose and care for AD/dementia patients. Moreover, different types of cameras along with different sensors and presence of detection technologies such as RFID, NFC, and Bluetooth can be used as a complement for overcoming the limitations of each technology and increasing the efficiency of monitoring systems. These technologies cover a wide range of dimensions of the patient's life and monitor all the psychological conditions, ADLs, and patient's sleep quantity and quality. Therefore, a personalized care program could be provided to the patients with AD/dementia based on their lifestyle and health status.

In general, these IoT technologies have some strengths and weaknesses, and their application for AD/dementia patients may increase their limitations; for example, the patient's agitation limits the use of technology, so that the technologies that monitor the patient's condition from greater distance appear to be more appropriate than those attached to the patient's body. In other words, cameras or sensors that are not attached to the body such as pressure sensors are superior to sensors embedded in bracelets or belts such as inertial sensors or GPS in movement recognition and tracking. Another issue with incorporating these technologies into IoT-based systems for monitoring Alzheimer's and dementia patients is that most systems presume that the patient is the only one who utilizes them; however, this is not always the case in practice and different persons may be present. A camera or smart ID card might be used to detect the presence of the real patient as a solution for this issue.

## Figures and Tables

**Table 1:** Inclusion and exclusion criteria.

Inclusion criteria	(1) Papers published in peer-reviewed journals and conferences, the full text of which was available (2) Studies that used the Internet of Things (IoT) for Alzheimer's disease (AD)/dementia (3) Studies with/without a control group (4) Studies that evaluated IoT interventions and reported the outcomes

Exclusion criteria	(1) Letters to the editor and editorials (2) Studies that only reported the technical specifications of a system (3) Studies that explicitly reported dementia occurring for a reason other than AD (4) Studies that focused on technological interventions other than IoT (5) Studies that used only global positioning system (GPS) or cameras (we included a study if it used a combination of GPS or camera with other IoT infrastructure) (6) Studies that only examined user satisfaction with the system or users' acceptance (we included studies, in which other outcomes in addition to satisfaction/acceptance, were reported)

**Table 2:** Typology of IoT technologies used for AD/dementia.

Type of technology	Objectives	Examples of applications	Some limitations
Sensors (36 studies)			

Inertial sensors (accelerometer, gyroscope, magnetometer) (17 studies) [20, 21, 23, 48, 53–65]	Recognizing physical activity and status of patients (standing, sitting, lying down, walking) [55, 61, 65]	(i) Using wearable sensors attached to the chest and shoes [54] or using them as a tag [20, 55], bracelet, and belt [59, 62] (ii) Diagnosing and predicting falls [20, 60, 62] (iii) Extracting movement patterns [62]	Current accelerometers do not specify place of performing activities at home [64]

Switch sensors (14 studies) [23, 26, 29, 47, 49, 55–57, 59, 62, 63, 66–68]	Installing switch sensors on doors and home appliances to monitor their use [49, 57, 66]	(i) Recognizing proper performance of activities and activity duration [23, 63, 68] (ii) Monitoring the entering and leaving to/from the living places [63, 68]	(i) Relatively high energy consumption when sending and displaying information on the monitors [68] (ii) Producing incorrect and repetitive information due to the entry and exit of people other than patients [49]

Sensors for vital signs (11 studies) [21, 23, 24, 26, 47, 58, 59, 63, 68–70]	Getting information about the patient's health including physical activities and vital signs [21, 23]	(i) Receiving data on blood pressure, body temperature, pulse, oxygen saturation, weight and hydration, daily step count and amount of physical activity, respiratory rate, and body movement, even during sleep [24] (ii) Using sensors in different ways including monitors installed at homes [24], wearable sensors [47, 63], sensors in belt [69], and sensors in mat [58] (iii) Recording data in sleep or awake modes [69, 70]	Possible impairment in estimating heart rate and breathing rhythm during sleep due to type of body movement in sleep [69, 70]

Pressure sensor (17 studies) [21, 23, 24, 26–28, 48, 50, 57–59, 63, 64, 67, 68, 71, 72]	Using sensors to measure body movement and monitoring sleep pattern [28, 58]	(i) Measuring bedtime, sleep duration and breaks, and sleep quality [21, 50, 67] (ii) Recognizing the person's agitation and wandering duration [68] (iii) Activating the alarm after the person getting out of bed [63, 72]	Misrecognizing the sleep mode when the person is calm and motionless in bed [71]

Infrared (IR) sensor (15 studies) [20, 22, 23, 26, 28, 50, 53, 55, 58, 62, 63, 66, 68, 71, 73]	Monitoring the places where the person is present during the day and night using activity recognition [63]	(i) Activity recognition [23, 55, 68, 73] (ii) Examining sleep patterns [50]	(i) Difficulty in recognizing the person's location when other people are present [55] (ii) Failure to record information when the person is motionless (e.g., watching TV in the bed) [58]

Cameras (18 studies)			

Red-green-blue-depth (RGB-d) camera (16 studies) [21–23, 29, 47, 53, 54, 57, 59, 60, 62, 67, 70, 72, 74, 75]	Depth cameras collect images and depth data and provide various data such as RGB, depth, infrared, patient skeleton movement recognition, and tracking data. Analyzing these data makes it possible to monitor patients' progress during rehabilitation exercises [29, 60, 70]	(i) Tracking the patients using a camera along with inertial sensors or IR [23] (ii) Depth cameras are better in predicting patient status than wearable sensors [21, 74] (iii) Identifying the person's movement direction using a variety of cameras and sensors [72]	Lack of enough accuracy for differentiating the activities related to the joints and small actions (e.g., eating and taking medication both involve the hand joints) [60]

Ordinary (cameras without sensors) (5 studies) [47, 59, 69, 71, 74]	Video camera is used at smart homes in different places [59]. This camera provides a panoramic view of the scene (360 degrees), which allows monitoring large indoor areas such as day-care centers [47, 59]	(i) Establishing a video surveillance system to prevent unwanted accidents for the patient during daily high-level activities such as cooking [47, 59] (ii) Improving the results of human tracking algorithms due to producing more comprehensive data by these types of devices [59]	A large volume of data is gathered by this type of camera, and a great amount of additional data is gathered by the recorded images [59]

Global positioning system (GPS) (7 studies) [23, 24, 27, 48, 50, 60, 61]	Tracking or positioning systems can indicate the location of the person with dementia [48, 61]	(i) Providing security for patients with dementia, especially when they suffer from agitation and depression [48] (ii) Easy access due to being used in various devices such as mobile phones [24] (iii) Ability to send location to caregivers via short message service (SMS) [24] (iv) Easy to use due to the small size of the devices [24, 61]	(i) GPS is not accurate enough indoors to locate the person [23] (ii) Using GPS for tracking is not suitable due to the error of about 8 m when a serious danger threatens a person in a path as they approach a certain point [23] (iii) High energy consumption of these devices and the need to charge the batteries daily (this limitation can be serious for people with dementia) [24, 50] (iv) If it is difficult for a person to use, fasten, and carry a GPS safety bracelet, they are reluctant to use it and, sometimes, remove it. Locked models are suggested in this case [48]

Communication technology			

Bluetooth (4 studies) [22, 50, 63, 68]	(i) Using Bluetooth technology to detect objects by tags that are enabled by proximity beacon [22, 68] (ii) Using this technology to transfer the gathered data to the main server [22, 50, 68]	(i) Possibility of installing positioning sensors on the walls along with Bluetooth tags attached to wheelchairs and patient equipment for tracking patients, especially when people with cognitive impairment disconnect wearable devices or those attached to the body [50]	

ZigBee (3 studies) [27, 55, 61]	(i) Using ZigBee to identify the person's location [27] (ii) Using this technology to make communication between the sensors around the person and central server [27]	(i) Appropriate accuracy using ZigBee along with IR sensors and combining the data of these two to locate the person [55]	(i) ZigBee is commonly used in conjunction with other technologies and most of the people are reluctant to carry multiple devices, so ZigBee application is limited [61] (ii) High energy consumption in ZigBee-equipped devices and the importance of improving battery life as a simple way to increase system reliability [55, 61]

Radio frequency identification (RFID) (1 study) [50]	Each active entity should be equipped with barcode-sized tags for unique identification. The data transmitter to the RFID server periodically retrieves the data from the tags and transmits them via Wi-Fi or Bluetooth [50]	(i) Increasing use of RFID in Internet of Things (IoT) to connect entities to the Internet [50]	

Near field communication (NFC) (1 study) [60]	Measuring devices containing NFC equipment are installed in fixed locations to determine the position and recognize activities [60].	(i) Installing an NFC tag on a specific place such as a dining table to sense the NFC tag installed on the medicine box and diagnose medicine consumption by the patient [60]	NFC should be used along with a camera to improve accuracy in recognizing activities [60]

**Table 3:** Results of using IoT technologies for monitoring purposes and its outcomes.

Aspects of care	Examples of positive or negative outcomes
Diagnosis (14 studies) [20, 26, 28, 29, 49, 50, 56, 57, 63–66, 73, 74]	+ Internet of Things (IoT) technology made it possible to monitor and compare the activity pattern of persons with mild cognitive impairment (MCI) with healthy elderly persons over a period of several months and diagnosed with Alzheimer's disease based on pattern changes [20, 66, 73]. + MCI can be diagnosed in a person using IoT based on how the computer is used [29, 56, 57]. + Early diagnosis of neurological disorders became possible by comparing the amount of activity during different periods using IoT [26, 29, 63, 66, 73]. + MCI can be diagnosed using IoT by comparing the daily activities of a healthy person with a patient with Alzheimer disease (AD), diagnosing the type of activities based on the person's presence in different places of the house, and comparing the results with those of conventional tests [20, 21, 26, 63, 73]. + MCI can be diagnosed by IoT-based machine learning techniques; e.g., in a study, random forest (RF) showed the best performance (precision-recall curve = 0.73, *F* − score = 0.77, sensitivity = 0.92) for this purpose [73]. + The outcomes of a scenario (carrying several tasks) had high correlation (*r* = 0.81) with the mini mental state examination (MMSE) score. Alzheimer's disease can be diagnosed with high accuracy by observing the activities through IoT (the area under the ROC (receiver operating characteristic) curve is =0.98, and the sensitivity and specificity are 100% and 94%). Monitoring tasks with IoT can assist in detecting MCI with high accuracy (the area under the ROC curve = 0.87, and the sensitivity and specificity are 74% and 89%) [29].

Activity of daily living (ADL) (27 studies) [20–22, 24, 26, 29, 47–50, 53–55, 57–63, 65, 67, 68, 71–73, 75]	+ IoT technology made it possible to extract behavioral patterns by examining the person's ADLs and recognizing unusual and dangerous behaviors [20, 54, 55, 57, 65, 67, 71]. + Using IoT technology improved daily activities such as ironing, cooking, and personal hygiene [47, 54, 62, 73]. + IoT technology made it possible to compare the two healthy and Alzheimer's groups in displacing kitchen utensils. The number of completed activities among people with Alzheimer's was significantly lower [29, 49, 53, 75]. + Using the IoT system, when employing a single sensor or multiple sensors, the mean *F*-measure for daily activity detection are high (*F*multiple = 81.2% and *F*mono = 88.2%) [53]. +From examining the degree of correlation between various activities by IoT system, obtained at night, there was 0.71 correlation between getting up and going to the bathroom. The correlation between waking up in the middle of the night and doing daily activities was -0.35, and the correlation between getting up at night and performing the usual activities of the elderly and medical staff was 0.47 [55]. + Activities could be precisely recognized by an IoT system. Sensitivity to identifying certain activities was reported as follows: cooking (84.29%), eating (87.78%), toileting (94.79%), and getting ready for bed (92.38%). Specificity of this system was reported as sleeping (85.77%), getting ready for bed (94.48%), eating (94.83%), seated activity (94.98%), and grooming (96.98%) [57].

Sleeping (19 studies) [20–23, 26, 28, 47, 48, 50, 55, 57, 58, 60, 62, 63, 67, 68, 72, 73]	+ Sleep monitoring using IoT is a good criterion for the early diagnosis of dementia [21, 23, 26, 28, 58, 67, 72]. + Using IoT showed that the sleep quality, quantity, and rhythm of a healthy person are better than that of a patient. Thus, IoT can be used to monitor sleep and diagnose AD [21, 26, 28, 58, 72]. + Using IoT made it possible to study changes in the sleep patterns of the elderly [23, 28, 47, 58]. + Using IoT improved patients' sleep duration and reduced frequent waking-up during the night [21, 58, 67]. + In monitoring sleeping by IoT system, the average sleep efficiency was found to be between 32.1% and 80% [58]. + Automatic IoT-based sleep analysis had similar findings to the individual's self-reports (sensitivity = 0.73, specificity = 0.88, accuracy = 0.85) [68]. + The participants' sleep patterns changed after the IoT intervention. For example: (i) Increased the deep sleep time from 7% to 38% (ii) Reduced the amount of time that spend awake at night (e.g., from 11 times to 6 times) (iii) Reduced light sleep from 74% to 58% of total sleep time (iv) Decreased the overall amount of time spent awake in bed at night from 3.79% to 22.1% [21] + The correlation between light sleep length and cognitive tests was 0.836, while the correlation between total sleep duration and cognitive tests was 0.843, according to sleep monitoring by an IoT system [47].

Medication (9 studies) [20, 21, 29, 47, 53, 60, 63, 67, 68]	+ IoT technology can monitor adherence to the medication regimen and gives the necessary alerts to healthcare providers or informal caregivers in the shortest time [20, 21, 29, 53, 67]. + Using IoT made it possible to monitor medication use and irregularities [47, 60, 63, 68].

Vital signs (11 studies) [21, 23, 24, 26, 47, 58, 59, 63, 68–70]	+ IoT technology enabled users to make alerts in the event of observing abnormalities in the patient's vital signs, along with a list of necessary actions in the case of encountering any alert [26, 47, 58, 63, 68]. + Using IoT and monitoring data such as heart rate or respiratory rate can predict agitation in AD/dementia patients [21, 24, 69, 70]. + IoT system is capable of measuring heart rate and respiratory rate (correlation between nurses and system was 0.874 without Charite Dome (ChD), 0.608 with ChD for heart rate, and 0.840 and 0.602 without ChD and with ChD for the respiratory rate [70].

Agitation (9 studies) [20–22, 24, 27, 61, 62, 69, 70]	+ IoT technology can reduce the stress and anxiety of the patient and their caregivers using warning messages appropriate to the patient's wandering and agitation state as well as severity of the danger that threatens them [24, 27, 69, 70]. + Acceptable results were obtained in using robots and systems equipped with IoT for helping the patient's wandering and agitation state [20, 22, 62]. + IoT system can detect persistent vocalizations by measuring the heart rate; it is found that the heart rate is around 40 beats per minute at times of persistent vocalizations [69].

Memory (5 studies) [47–49, 63, 73]	+ IoT can remind people of different types of activities and reduce dependence on others to help them remember to do ADLs correctly [47, 63, 73]. + The effect of using IoT technology on helping the independence and security of people with memory impairment showed patients' independence in performing activities such as daily walking [48, 49, 63]. + Correlation between traditional memory test (face name test) and an IoT system was 0.597, whereas the correlation between response time and face name test was 0.341. Therefore, IoT system can measure memory abilities in AD/dementia [49].

Social interaction (4 studies) [21, 26, 63, 67]	+ Accurate and continuous monitoring of social interactions using IoT showed individuals with better social interactions obtained higher scores in executive function tests [21, 26, 67]. + Using IoT can help better understand the relationship between social interactions and cognitive decline [21, 63, 67]. + IoT reduced depression, isolation symptoms, and watching television as well as increased social interactions with others and participation in various social programs [26, 63, 67].

Apathy (3 studies) [20, 59, 62]	+ Behavioral patterns of patients can be examined to monitor normal and abnormal behaviors as a sign of apathy in individuals using the data obtained from the IoT-enabled systems [20, 59, 62]. + IoT-based systems can differentiate normal and abnormal behaviors accurately (accuracy = 98.4%, precision = 98.7%, recall = 98.3%) [59].

Movement (23 studies) [20–22, 29, 47, 48, 50, 53–55, 57, 58, 60, 62–65, 67, 68, 70, 72–74]	+ Using IoT for continuously assessing walking speed at home provided a better understanding of changes in people's speed over time [22, 55, 58, 62]. + Walking speed and its daily variability obtained using IoT can be among the first symptoms of MCI progression [21, 62, 64, 65]. + Movement disorders and disability in ADLs, MCI, and dementia caused by AD can be diagnosed at an earlier time using sensor technology and gait analysis [55, 62, 64, 65, 68, 74]. - Walking in the home environment was generally at low acceleration and rarely in a straight line; therefore, it is difficult to develop algorithms that use accelerometers to detect changes in walking [72, 73]. + An IoT-based machine learning method can detect sitting, sleeping, or standing positions accurately (97% accuracy by Naive Bayes (NB) and 99.1% accuracy by support vector machine (SVM) [60]. + By using IoT system and detection of movements, it is possible to predict MCI and Alzheimer's disease. For example, after 3.2 years, 25% of persons were diagnosed by MCI, and two of the 12 movements previously detected by IoT system were useful in predicting MCI. This figure for Alzheimer's was 9.4% of the participants using four types of movements [65].

Tracking (12 studies) [21, 23, 24, 27, 48, 50, 54, 55, 59–62]	+ Patients who go out of the safe area could be identified using IoT, and their location could be tracked and informed to their caregivers using mobile phones [24, 50, 61]. + An IoT-based monitoring system for the detection of patient's presence yielded positive results, as follows: (i) The average detection time of patient's departure from the safe region was 1.97 sec, which was enough to make a notification [61] (ii) In 82% of the cases, the presence of a patient could be detected at the distance of 48-55 m [61]

Fall (8 studies) [21, 24, 48, 59, 60, 62, 68, 72]	+ IoT could reduce the risk of falling [24, 48, 72]. + Probability of falling was reduced in the intervention group (*p* = 0.034) using an IoT system [72].

+ indicates positive outcomes, and – indicates negative outcomes.

**Table 4:** Technology evaluations.

Technology evaluation	Examples of evaluation results	Aspects of care
Feasibility (3 studies) [54, 55, 71]	+ It is feasible to identify relationship among some events that are difficult for the treatment team to observe without using IoT-based monitoring system [55]	Activities of daily living (ADL), sleeping, movement, tracking
	- Feeling uncomfortable while doing activities [71]	ADL
	- Long-term monitoring scenarios were undesirable [71]	ADL
	- Difficulty in performing some activities using IoT technologies such as retrieving objects or making a phone call [71]	ADL

Usability (3 studies) [23, 48, 61]	+ The caregivers usually indicate the usefulness of the system in the following cases: (i) Tracking patients by location monitoring systems [48, 61] (ii) Finding out which locations are dangerous for patients [48, 61] (iii) Taking necessary measures for patients [23]	ADL, sleeping, agitation, memory, movement, tracking, fall, vital signs
	- Global positioning system (GPS), despite being available in a price range suitable for people at different levels, is not widely used in practice, except in clinical studies [23]	Tracking

Acceptability (3 studies) [54, 55, 57]	+ Given that collecting some patients' data is not possible with current care methods such as distance traveled, routes within the care unit, time spent in each area, wandering, and waking up at night, this technology is acceptable by the medical team [55]	ADL, sleeping, movement, tracking
	+ Daily activity monitoring system is acceptable for patients [57]	Diagnosis, ADL, sleeping
	+ This technology is considered desirable by many users [54]	ADL, tracking, movement
	- 67% of the participants were willing to be evaluated by the sensor at the place of residence only for a limited time during the day (not for long duration) [54]	ADL, tracking, movement

+ indicates positive results, and – indicates negative results.

## Supplementary Materials

Supplementary 1 Supplementary data 1 includes PRISMA Extension for Scoping reviews (PRISMA-ScR) 2018 Checklist.

Supplementary 2 Supplementary data 2 includes search strategy, characteristics of included studies, and findings on the use of the Internet of Things in Alzheimer's disease.
