# Supplementary material for: Applications and Outcomes of Internet of Things for Patients with Alzheimer's Disease/Dementia: A Scoping Review
Source: Biomed Res Int. 2022 Mar 15;2022:6274185. doi: 10.1155/2022/6274185 (PMC8948545; doi:10.1155/2022/6274185)
Supplement: Supplementary 1 — Supplementary data 1 includes PRISMA Extension for Scoping reviews (PRISMA-ScR) 2018 Checklist. [file 6274185.f1.docx]

**Supplementary data 1**

PRISMA Extension for Scoping reviews (PRISMA-ScR) 2018 Checklist

Table S1. PRISMA Extension for Scoping reviews (PRISMA-ScR) 2018 Checklist

| Section/topic | # | PRISMA-ScR Checklist item | Reported on article# |
| --- | --- | --- | --- |
| TITLE | | |  |
| Title | 1 | Identify the report as a scoping review. | Title |
| ABSTRACT | | |  |
| Structured summary | 2 | Provide a structured summary ;, as applicable: background; objectives; data sources; study eligibility criteria, participants, and interventions; study synthesis methods; results; limitations; conclusions and implications of key findings. | Abstract |
| INTRODUCTION | | |  |
| Rationale | 3 | Describe the rationale for the review in the context of what is already known. Explain why the review question(s)/objective(s) lend themselves to a scoping review approach. | Section 1, paragraph1-5, page 3-4 |
| Objectives | 4 | Provide an explicit statement of the question(s) and objective(s) being addressed with reference to their key elements (e.g., population or participants, concepts and context), or other relevant key elements used to conceptualize the review question(s) and/or objective(s)). | Section 1, paragraph 6,  page 4 |
| METHODS | | |  |
| Protocol and registration | 5 | Indicate if a review protocol exists, if and where it can be accessed (e.g., Web address), and, if available, provide registration information including registration number. | This review was registered in Iran University of Medical Sciences (code: 96-04-136-32546). |
| Eligibility criteria | 6 | Specify the characteristics of the sources of evidence (e.g., years considered, language, publication status) used as criteria for eligibility, and provide a rationale. | Section 2.2 Table1, page 8 |
| Information sources | 7 | Describe all information sources (e.g., databases with dates of coverage, contact with study authors to identify additional sources) in the search and date last searched. | Section 2.1, page 7-8 |
| Search | 8 | Present full electronic search strategy for at least one database, including any limits used, such that it could be repeated. | Section 2.1, page 7-8  and  Table S1 in Supplementary data 2 |
| Selection of sources of evidence | 9 | State the process for selecting studies (i.e., screening, eligibility) included in the scoping review. | Section 2.2 Table 1,  page 8 |
| Data charting process | 10 | Describe the methods of charting data from the included sources of evidence (e.g. piloted forms; forms that have been tested by the team before their use, whether data charting was done independently, in duplicate) and any processes for obtaining and confirming data from investigators. | Section 2.3, page 9 |
| Data items | 11 | List and define all variables for which data were sought and any assumptions and simplifications made. | Section 2.3, page 9 |
| Critical appraisal of individual sources of evidence | 12 | **If done,** provide a rationale for conducting a critical appraisal of included sources of evidence; describe the methods used and how this information was used in any data synthesis (if appropriate). | NA |
| Summary measures | 13 | Not applicable for scoping reviews. | NA |
| Synthesis of results | 14 | Describe the methods of handling and summarizing the data that were charted. | Section 2.3, page 9 |
| Risk of bias across studies | 15 | Not applicable for scoping reviews. | NA |
| Additional analyses | 16 | Not applicable for scoping reviews. | NA |
| RESULTS | | |  |
| Selection of sources of evidence | 17 | Give numbers of studies screened, assessed for eligibility, and included in the review, with reasons for exclusions at each stage, ideally using a flow diagram. | Section 3.1, Fig 1, page 9-10 |
| Characteristics of sources of evidence | 18 | For each source of evidence, present characteristics for which data were charted and provide the citations. | Table S2 and Table S3 in Supplementary data 2 |
| Critical appraisal within sources of evidence | 19 | If done, present data on critical appraisal of included sources of evidence (see item 12). | NA |
| Results of individual sources of evidence | 20 | For each included source of evidence, present the relevant data that were charted that relate to the review question(s) and objective(s). | Table S3 in Supplementary data 2 |
| Synthesis of results | 21 | Summarize and/or present the charting results as they relate to the review question(s) and objective(s). | Section 3.5, Table 2, Section 3.6, Table 3, Section 3.7, Table 4,  page 13-22  Table S3 in Supplementary data 2 |
| Risk of bias across studies | 22 | Not applicable for scoping reviews. | NA |
| Additional analysis | 23 | Not applicable for scoping reviews. | NA |
| DISCUSSION | | |  |
| Summary of evidence | 24 | Summarize the main results (including an overview of concepts, themes, and types of evidence available), explain how they relate to the review question(s) and objectives, and consider the relevance to key groups | Section 4, page 23-29 |
| Limitations | 25 | Discuss the limitations of the scoping review process. | Section 5, page 28 |
| Conclusions | 26 | Provide a general interpretation of the results with respect to the review question(s) and objective(s), as well as potential implications and/or next steps. | Section 7, page 29-30 |
| FUNDING | | |  |
| Funding | 27 | Describe sources of funding for the included sources of evidence, as well as sources of funding for the scoping review. Describe the role of the funders of the scoping review. | Page 30 |
