# Supplementary material for: Applications and Outcomes of Internet of Things for Patients with Alzheimer's Disease/Dementia: A Scoping Review
Source: Biomed Res Int. 2022 Mar 15;2022:6274185. doi: 10.1155/2022/6274185 (PMC8948545; doi:10.1155/2022/6274185)
Supplement: Supplementary 2 — Supplementary data 2 includes search strategy, characteristics of included studies, and findings on the use of the Internet of Things in Alzheimer's disease. [file 6274185.f2.docx]

**Supplementary data 2**

**Content**

Table S1. Strategy search in selected databases

Table S2. Characteristics of included studies

Table S3: Summary of findings on the use of the Internet of Things in Alzheimer's disease

Table S1: Strategy search in selected databases

| Cochrane | (“Alzheimer”:ti,ab,kw or “Dementia”:ti,ab,kw) and (“internet of things”:ti,ab,kw or “IoT”: ti,ab,kw or “RFID”: ti,ab,kw or “radio frequency identification”: ti,ab,kw or “monitoring physiologic”:ti,ab,kw or “WSN”: ti,ab,kw or “wireless sensor network”: ti,ab,kw or “wireless sensor system”: ti,ab,kw or “WBAN”: ti,ab,kw or “wireless body area network”: ti,ab,kw or “HAN”: ti,ab,kw or “human area network”: ti,ab,kw or “Wi-Fi”: ti,ab,kw or “ZigBee”: ti,ab,kw or “Bluetooth”: ti,ab,kw or “NFC”: ti,ab,kw or “near field communication”: ti,ab,kw or “Sensor”: ti,ab,kw or “wearable health monitoring”: ti,ab,kw or “wearable sensor”: ti,ab,kw or “sensor technologies”: ti,ab,kw or “remote patient monitoring”: ti,ab,kw or “smart health monitoring”: ti,ab,kw or “ubiquitous computing”: ti,ab,kw or “intelligent systems patient monitoring”: ti,ab,kw or “motion sensor”: ti,ab,kw or “GPS”: ti,ab,kw or “GSM”: ti,ab,kw or “GPRS”: ti,ab,kw or “pervasive computing”: ti,ab,kw or “distributed computing”: ti,ab,kw or “smart environments”: ti,ab,kw or “human activity recognition”: ti,ab,kw or “machine to machine communications”: ti,ab,kw or “m2m communications”: ti,ab,kw or “physical activity monitoring”: ti,ab,kw or “monitoring System”: ti,ab,kw or “patient monitoring”: ti,ab,kw or “physiological monitoring”: ti,ab,kw or “physiologic monitoring”: ti,ab,kw or “cloud processing”: ti,ab,kw or “cloud storage” ”:ti,ab,kw or “cloud service”: ti,ab,kw or “assistive technology”: ti,ab,kw or “self-help device”: ti,ab,kw or :assistive device”: ti,ab,kw or “Actigraphy”: ti,ab,kw or “web of things”:ti,ab,kw or “inertial sensor”: ti,ab,kw or “assisted living”: ti,ab,kw or “Accelerometer”: ti,ab,kw or “wearable technology”: ti,ab,kw or “motion sensing”: ti,ab,kw or “smart ambient assisted living”: ti,ab,kw or “Telehealth”: ti,ab,kw or “home monitoring”: ti,ab,kw or “smart sensor”: ti,ab,kw or “predictive monitoring”: ti,ab,kw or “monitoring technology”: ti,ab,kw or “smart device”) Publication Year from 1997 to 2021 (Word variations have been searched) |
| --- | --- |
| Embase | (Alzheimer OR Dementia) AND (“internet of things” OR IoT OR RFID OR “radio frequency identification” OR “monitoring physiologic” OR (WSN) OR “wireless sensor network” OR “wireless sensor system” OR WBAN OR “wireless body area network” OR HAN OR “human area network” OR Wi-Fi OR ZigBee OR Bluetooth OR NFC OR “near field communication” OR Sensor OR “wearable health monitoring” OR “wearable sensor” OR “sensor technologies” OR “remote patient monitoring” OR “smart health monitoring” OR “ubiquitous computing” OR “intelligent systems patient monitoring” OR “motion sensor” OR GPS OR GSM OR GPRS OR “pervasive computing” OR “distributed computing” OR “smart environments” OR “human activity recognition” OR “machine to machine communications” OR “m2m communications” OR “physical activity monitoring” OR “monitoring System” OR “patient monitoring” OR “physiological monitoring” OR “physiologic monitoring” OR “cloud processing” OR “cloud storage” OR “cloud service” OR “assistive technology” OR “self-help device” OR “assistive device” OR Actigraphy OR “web of things” OR “inertial sensor” OR “assisted living” OR Accelerometer OR “wearable technology” OR “motion sensing” OR “smart ambient assisted living” OR Telehealth OR “home monitoring” OR “smart sensor” OR “predictive monitoring” OR “monitoring technology” OR “smart device” |
| IEEE | (("Index Terms”: Alzheimer OR "Index Terms”: Dementia) AND ("Index Terms”: “internet of things “OR "Index Terms”: IoT OR "Index Terms": RFID OR "Index Terms”: “radio frequency identification” OR "Index Terms": “monitoring physiologic” OR "Index Terms": WSN OR "Index Terms”: “wireless sensor network” OR "Index Terms”: “wireless sensor system” OR "Index Terms": WBAN OR "Index Terms”: “wireless body area network” OR "Index Terms”: HAN OR "Index Terms”: “human area network” OR "Index Terms”: Wi-Fi OR "Index Terms”: ZigBee OR "Index Terms": Bluetooth OR "Index Terms": NFC OR "Index Terms": “near field communication” OR "Index Terms": Sensor OR "Index Terms": “wearable health monitoring” OR "Index Terms": “wearable sensor” OR "Index Terms": “sensor technologies” OR "Index Terms": “remote patient monitoring” OR "Index Terms": “smart health monitoring” OR "Index Terms": “ubiquitous computing” OR "Index Terms": “intelligent systems patient monitoring” OR "Index Terms": “motion sensor” OR "Index Terms": GPS OR "Index Terms": GSM OR "Index Terms": GPRS OR "Index Terms": “pervasive computing” OR "Index Terms": “distributed computing” OR "Index Terms": “smart environments” OR "Index Terms": “human activity recognition” OR "Index Terms": “machine to machine communications” OR "Index Terms":“m2m communications” OR "Index Terms": “physical activity monitoring” OR "Index Terms": “monitoring System” OR "Index Terms": “patient monitoring” OR "Index Terms": “physiological monitoring” OR "Index Terms": “physiologic monitoring” OR "Index Terms": “cloud processing” OR "Index Terms": “cloud storage” OR "Index Terms": “cloud service” OR "Index Terms": “assistive technology” OR "Index Terms": “self-help device” OR "Index Terms": “assistive device” OR Actigraphy OR "Index Terms": “web of things” OR "Index Terms": “inertial sensor” OR "Index Terms": “assisted living” OR Accelerometer OR "Index Terms": “wearable technology” OR "Index Terms": “motion sensing” OR "Index Terms": “smart ambient assisted living” OR Telehealth OR "Index Terms": “home monitoring” OR "Index Terms": “smart sensor” OR "Index Terms": “predictive monitoring” OR "Index Terms": “monitoring technology” OR "Index Terms": “smart device”)) |
| Ovid | ((Alzheimer OR Dementia) AND (“internet of things” OR IoT OR RFID OR “radio frequency identification” OR “monitoring physiologic” OR WSN OR “wireless sensor network” OR “wireless sensor system” OR WBAN OR “wireless body area network” OR HAN OR “human area network” OR Wi-Fi OR ZigBee OR Bluetooth OR NFC OR “near field communication” OR Sensor OR “wearable health monitoring” OR “wearable sensor” OR “sensor technologies” OR “remote patient monitoring” OR “smart health monitoring” OR “ubiquitous computing” OR “intelligent systems patient monitoring” OR “motion sensor” OR GPS OR GSM OR GPRS OR “pervasive computing” OR “distributed computing” OR “smart environments” OR “human activity recognition” OR “machine to machine communications” OR “m2m communications” OR “physical activity monitoring” OR “monitoring System” OR “patient monitoring” OR “physiological monitoring” OR “physiologic monitoring” OR “cloud processing” OR “cloud storage” OR “cloud service” OR “assistive technology” OR “self-help device” OR “assistive device” OR Actigraphy OR “web of things” OR “inertial sensor” OR “assisted living” OR Accelerometer OR “wearable technology” OR “motion sensing” OR “smart ambient assisted living” OR Telehealth OR “home monitoring” OR “smart sensor” OR “predictive monitoring” OR “monitoring technology” OR “smart device”)) |
| PubMed | (((Alzheimer[Title/Abstract] OR Dementia[Title/Abstract]) AND (“internet of things”[Title/Abstract] OR IoT[Title/Abstract] OR RFID[Title/Abstract] OR “radio frequency identification”[Title/Abstract] OR “monitoring physiologic” [Title/Abstract] OR WSN[Title/Abstract] OR “wireless sensor network” [Title/Abstract] OR “wireless sensor system” [Title/Abstract] OR WBAN[Title/Abstract] OR “wireless body area network” [Title/Abstract] OR HAN[Title/Abstract] OR “human area network” [Title/Abstract] OR Wi-Fi[Title/Abstract] OR ZigBee[Title/Abstract] OR Bluetooth[Title/Abstract] OR NFC[Title/Abstract] OR “near field communication” [Title/Abstract] OR Sensor[Title/Abstract] OR “wearable health monitoring” [Title/Abstract] OR “wearable sensor” [Title/Abstract] OR “sensor technologies” [Title/Abstract] OR “remote patient monitoring” [Title/Abstract] OR “smart health monitoring” [Title/Abstract] OR “ubiquitous computing” [Title/Abstract] OR “intelligent systems patient monitoring” [Title/Abstract] OR “motion sensor” [Title/Abstract] OR GPS[Title/Abstract] OR GSM[Title/Abstract] OR GPRS[Title/Abstract] OR “pervasive computing” [Title/Abstract] OR “distributed computing” [Title/Abstract] OR “smart environments” [Title/Abstract] OR “human activity recognition” [Title/Abstract] OR “machine to machine communications” [Title/Abstract] OR “m2m communications” [Title/Abstract] OR “physical activity monitoring” [Title/Abstract] OR “monitoring System” [Title/Abstract] OR “patient monitoring” [Title/Abstract] OR “physiological monitoring” [Title/Abstract] OR “physiologic monitoring” [Title/Abstract] OR “cloud processing” [Title/Abstract] OR “cloud storage” [Title/Abstract] OR “cloud service” [Title/Abstract] OR “assistive technology” [Title/Abstract] OR “self-help device” [Title/Abstract] OR “assistive device” [Title/Abstract] OR Actigraphy[Title/Abstract] OR “web of things” [Title/Abstract] OR “inertial sensor” [Title/Abstract] OR “assisted living” [Title/Abstract] OR Accelerometer[Title/Abstract] OR “wearable technology” [Title/Abstract] OR “motion sensing” [Title/Abstract] OR “smart ambient assisted living” [Title/Abstract] OR Telehealth[Title/Abstract] OR “home monitoring” [Title/Abstract] OR “smart sensor” [Title/Abstract] OR “predictive monitoring” [Title/Abstract] OR “monitoring technology” [Title/Abstract] OR “smart device” [Title/Abstract])) AND (“1997/01/01”[PDAT]:”2020/12/31”[PDAT])) |
| Scopus | ((TITLE-ABS-KEY (Alzheimer) OR TITLE-ABS-KEY (Dementia)) AND (TITLE-ABS-KEY (“internet of things”) OR TITLE-ABS-KEY (IoT) OR TITLE-ABS-KEY (RFID) OR TITLE-ABS-KEY (“radio frequency identification”) OR TITLE-ABS-KEY (“monitoring physiologic”) OR TITLE-ABS-KEY (WSN) OR TITLE-ABS-KEY (“wireless sensor network”) OR TITLE-ABS-KEY (“wireless sensor system”) OR TITLE-ABS-KEY (WBAN) OR TITLE-ABS-KEY (“wireless body area network”) OR TITLE-ABS-KEY (HAN) OR TITLE-ABS-KEY (“human area network”) OR TITLE-ABS-KEY (Wi-Fi) OR TITLE-ABS-KEY (ZigBee) OR TITLE-ABS-KEY (Bluetooth) OR TITLE-ABS-KEY (NFC) OR TITLE-ABS-KEY (“near field communication”) OR TITLE-ABS-KEY (Sensor) OR TITLE-ABS-KEY (“wearable health monitoring”) OR TITLE-ABS-KEY (“wearable sensor”) OR TITLE-ABS-KEY (“sensor technologies”) OR TITLE-ABS-KEY (“remote patient monitoring”) OR TITLE-ABS-KEY (“smart health monitoring”) OR TITLE-ABS-KEY (“ubiquitous computing”) OR TITLE-ABS-KEY (“intelligent systems patient monitoring”) OR TITLE-ABS-KEY (“motion sensor”) OR TITLE-ABS-KEY (GPS) OR TITLE-ABS-KEY (GSM) OR TITLE-ABS-KEY (GPRS) OR TITLE-ABS-KEY (“pervasive computing”) OR TITLE-ABS-KEY (“distributed computing”) OR TITLE-ABS-KEY (“smart environments”) OR TITLE-ABS-KEY (“human activity recognition”) OR TITLE-ABS-KEY (“machine to machine communications”) OR TITLE-ABS-KEY (“m2m communications”) OR TITLE-ABS-KEY (“physical activity monitoring”) OR TITLE-ABS-KEY (“monitoring System”) OR TITLE-ABS-KEY (“patient monitoring”) OR TITLE-ABS-KEY (“physiological monitoring”) OR TITLE-ABS-KEY (“physiologic monitoring”) OR TITLE-ABS-KEY (“cloud processing”) OR TITLE-ABS-KEY (“cloud storage”) OR TITLE-ABS-KEY (“cloud service”) OR TITLE-ABS-KEY (“assistive technology”) OR TITLE-ABS-KEY (“self-help device”) OR TITLE-ABS-KEY (“assistive device”) OR TITLE-ABS-KEY (Actigraphy) OR TITLE-ABS-KEY (“web of things”) OR TITLE-ABS-KEY (“inertial sensor”) OR TITLE-ABS-KEY (“assisted living”) OR TITLE-ABS-KEY (Accelerometer) OR TITLE-ABS-KEY (“wearable technology”) OR TITLE-ABS-KEY (“motion sensing”) OR TITLE-ABS-KEY (“smart ambient assisted living”) OR TITLE-ABS-KEY (Telehealth) OR TITLE-ABS-KEY (“home monitoring”) OR TITLE-ABS-KEY (“smart sensor”) OR TITLE-ABS-KEY (“predictive monitoring”) OR TITLE-ABS-KEY (“monitoring technology”) OR TITLE-ABS-KEY (“smart device”))) |
| Web of Science | (((TI= ((Alzheimer disease) OR (Alzheimer’s disease) OR (Alzheimer dementia) OR (Alzheimer sclerosis) OR (Alzheimer syndrome) OR (presenile Alzheimer dementia) OR (early onset Alzheimer disease) OR (Alzheimer disease AND early onset) OR (familial Alzheimer disease) OR (focal onset Alzheimer’s disease) OR (Alzheimer’s disease AND focal onset) OR (late onset Alzheimer disease) OR (Alzheimer disease AND late onset) OR (presenile dementia) OR (dementia AND presenile) OR (senile dementia AND acute confusional) OR (acute confusional senile dementia) OR (dementia AND primary senile degenerative) OR (Alzheimer type senile dementia) OR (senile dementia AND Alzheimer type) OR (dementia AND Alzheimer type) OR (senile dementia) OR (dementia AND senile) OR (primary senile degenerative dementia) OR (Alzheimer type dementia) OR (dementia AND Alzheimer-type) OR (Alzheimer-type dementia) OR (dementia AND Alzheimer) OR (syndrome AND Alzheimer) OR (sclerosis AND Alzheimer) OR (disease AND Alzheimer) OR (Alzheimer Disease/physiopathology) OR (Alzheimer Disease/prevention and control) )) AND )TS=(internet of things) OR TOPIC: (IoT) OR TOPIC: (internet of controlled things) OR TOPIC: (RFID) OR TOPIC: (radio frequency identification) ORTOPIC: (monitoring physiologic) OR TOPIC: (self-help devices) OR TOPIC: (WSN) OR TOPIC: (wireless sensor network) OR TOPIC: (WBAN) OR ("near field communication") OR TOPIC: ("human area network") OR TOPIC: (Wi-Fi) OR TOPIC: (Zigbee) OR TOPIC: (Bluetooth) OR TOPIC: (NFC) OR (Sensor OR wearable health monitoring OR wearable sensor OR sensor technologies OR remote patient monitoring OR smart health monitoring OR ubiquitous computing OR intelligent systems patient monitoring OR motion sensor OR GPS OR GSM OR GPRS OR pervasive computing OR distributed computing OR smart environments OR human activity recognition OR machine to machine communications OR m2m communications OR physical activity monitoring OR monitoring System OR (monitoring AND physiologic) OR patient monitoring OR (monitoring AND physiological) OR physiological monitoring OR physiologic monitoring OR (monitoring AND patient) OR (computing AND cloud) OR cloud processing OR (processing AND cloud) OR cloud storage OR (storage AND cloud) OR (storages AND cloud) OR cloud service OR (service AND cloud) OR assistive technology OR assistive technologies OR (device AND self-help) OR (devices AND self-help) OR self-help device OR (technologies AND assistive) OR (technology AND assistive) OR assistive devices OR assistive device OR (device AND assistive) OR (devices AND assistive) OR Actigraphy OR web of things OR inertial sensor OR assisted living OR Accelerometer OR wearable technology OR motion sensing OR smart homes OR smart home OR ambient assisted living OR Telehealth OR home monitoring OR smart sensor OR smart sensors OR predictive monitoring OR monitoring technology OR monitoring technologies OR smart device OR smart devices)) AND YEAR PUBLISHED: (1997-2020)) |

Table S2: Characteristics of included studies (ordered by year)

| # | Author/Year/Country/Reference | Purpose | Intervention | Participants | Intervention duration | Technology |
| --- | --- | --- | --- | --- | --- | --- |
| 1 | Franco/2010/France[26] | Early detection of dementia utilizing 24h monitoring of patients using the Telehealth Care program to improve patients’ quality of life | Developing a monitoring environment using a number of devices.  Using a variety of sensors to monitor sleep, waking up time, and other daily activities  Keeping a record of how much and what kind of activities participants performed during every time period.  Using vital sensor to measure body temperature, sweating rate.  In the case of a major deviation in a person's pattern, sending an alert signal to his\her caregivers. | A 80 years old woman who lives alone. | 6 months | A variety of sensor types (IR, switch, pressure, sensors for vital signs) |
| 2 | Wang/2010/  England[28] | Monitoring sleep patterns (quantity and quality) in healthy older people and dementia for early dementia diagnosis. | Sensors are placed throughout the sleeping area. Obtaining information regarding sleep status and sleep episodes to check the conditions in or outside the bed. | 1 Alzheimer's patient and 1 healthy male with occasional memory loss | 1 month | IR sensor, pressure sensor |
| 3 | Taub/2011/  USA[27] | Avoiding issues that cause the patient to wander. | The Escort system prepares the person's location information in the chosen area and generates and sends an alert to patient's caregivers.  The patient wears a little wearable token that can be used to both find the individual and connect with the central server automatically. To communicate, it uses ZigBee technology. Additionally, the person's location information is manually collected and recorded. | 367 alerts were stored in the database after patients were separated into groups of two to four. In separate work shifts, six patient caregivers were participated. | 12 weeks | ZigBee, GPS, pressure sensors |
| 4 | Sacco/2012/  France[29] | Investigating the feasibility of utilizing a video surveillance system to evaluate everyday activities in persons with Alzheimer's disease and mild cognitive impairment on a quantitative and qualitative basis. | It was evaluated how to complete the tasks required by the person using an observation room that employs a variety of sensors and cameras equipped with sensors to recognize the person's presence and activity. | 108 individuals:  Protocol 1: 60 Alzheimer's patients with an average age of 76.7 and an average MMSE score of 20.7, plus 10 healthy people with an average age of 73.9 and an average MMSE score of 28.1.  Protocol 2: 19 adults with mild cognitive impairment, average age 75.2, average MMSE score 25.8, and 19 healthy people, average age 71.1, average MMSE score 28.8. | Less than a day | Camera equipped with sensors, switch sensor |
| 5 | Crispim/2013/France[53] | By integrating sensor data and comparing the diagnosis of individual daily activities across two modes of usage of single sensor and multiple sensor, a framework for detecting events relevant to the daily activities of older persons is being developed. | In a hospital room equipped with home appliances, people's activities were monitored using cameras and sensors.  A special alert was sent to the caregivers when the activities were recognized, based on the significance of each activity. | Unknown number, all over 65 years old, including some with Alzheimer's disease. Some were in the control group as well (healthy individuals in terms of cognitive and behavioral disorders). | Less than a day | Camera equipped with a sensor, IR sensor  One wearable inertial sensor and 2 video cameras as input sensors |
| 6 | Robert/2013/France[54] | Developing a novel framework for modeling daily activities. | Installing video and audio sensors in a special room.  Using technologies in this room to assess activities of daily living. The room was equipped with armchair, workstation, tea room, TV, PC, library, and 2D camera to conduct daily activities and IADL.  the patients were asked to conduct a series of predetermined activities.  For outdoor activities, an accelerometer sensor was also used. | 16 persons with mild to severe Alzheimer's disease (mean age 76.7 and mean MMSE score of 20.7) and 10 elderly people who were cognitively well participated in the first portion of the study. (Mean age is 73.9, and the MMSE score is 28.1)  For the feasibility study, there were 64 people in total: 22 healthy people, 30 MCI patients, and 12 Alzheimer's patients. | Less than a day | Camera equipped with sensors and a variety of audio and video sensors and presence detection and accelerometer sensors |
| 7 | Chan/2014/ China[55] | Developing a smart monitoring system that can provide alerts when a person's usual lifestyle is violated. | Collecting people's movement data and providing normal or abnormal alerts to their caregivers immediately, in the event of a dangerous situation or behavioral abnormality.  Using eight infrared sensors in the apartment's bedroom and bathroom.  A tag was put on person clothing and when the person's position was close to the rays, their position was recorded. An accelerometer, a microprocessor, and a ZigBee wireless receiver module are part of the system. | 4 patients and medical personnel | 16 days | ZigBee, IR sensor, switch sensor, inertial sensor(accelerometer) |
| 8 | Kaye/2014/ USA[56] | Diagnosing mild cognitive impairment and determining whether long-term changes in computer use (remotely monitored) differentiate between healthy and cognitively impaired patients. | Data was gathered from 230,000 computer sessions. Participants were given cognitive tests before a home sensor network was set up to track their activities, such as usual ADL, time away from home, and walking speed.  In the final phase, each person's computer settings and relevant training were completed. Individual activities when interacting with the system were captured using a computer algorithm. | There were 113 patients in total (38 MCI patients and 75 healthy patients) | 36 months | Switch sensors, inertial sensor(accelerometer) |
| 9 | Stucki/2014/ Switzerland [57] | Providing a passive assistive system for identifying and classifying daily activities that is web-based, and non-invasive. | Participants were required to maintain a log of their daily activities for a period of 20 days. Each individual is given a device with ten switches, and when they go to perform any task, they turn on the corresponding switch. A wearable switch is also included with the device. | There are 11 people in all, one of whom has dementia (84 years old, MoCA test score equal to 23)  As a control group, ten healthy people participated (mean age 48 years, and MoCA score between 27 to 30) | 6 months | Camera equipped with inertial sensor, pressure sensor, switch sensor |
| 10 | Xin zhu/2014/ Japan[58] | To monitor the sleep quality of nursing home residents over time using an Internet-based automated sleep monitoring system (Umemory) | A piezoelectric sensor, a peripheral sensor box, a bedside box for data transmission, and a central server at another location compensate the sleep monitoring system.  Sensors detected in-bed and out-of-bed situations. Sensors were used to collect data on heart rate, breathing rate, and body movements during sleeping. | 13 persons in all. 10 (dementia patients) are all females, with an average age of 82.8 years. | 1090 days | IR sensor, Sensors for vital signs, pressure sensor, inertial sensor (accelerometer)  Using a vital sensor to measure heart rate, and respiration rate |
| 11 | Ahmad Akl/2015/  USA[66] | Diagnosing MCI in adults automatically, based on indicators provided by sensor technology, signal processing, and machine learning. | Implementation of various sensors in apartments (non­invasive sensing technology), contact switches at the entrance, and Motion sensor on the ceiling.  A questionnaire was also used to collect more information. | 97 persons in total. 70% of them were mentally healthy and lived independently. | More than three years | IR sensor, switch sensors |
| 12 | KaraKostas/  2015/  Greece[67] | Providing a sensor-based system to assist Alzheimer's patients in their daily living, and study the feasibility of doing effective treatment interventions for patients using this system. | The proposed system monitors patients at home and records daily activities. Data is collected by various sensors, and a component provides patient assistance by sending data to patient carers. Patient support was provided with unique interventions based on the information gathered through the system. | one 79-year-old woman who lives alone. | 3 months | Camera equipped with RGB-D sensor, switch sensor, pressure sensor |
| 13 | Jekel/2016/  Germany[71] | To investigate the potential of a smart home environment for IADL assessment in MCI patients. | Healthy and patients with MCI were asked to do six activities in a smart home with two rooms equipped with sensors and cameras. Each of these six actions is broken down into multiple steps that can be tracked more closely. | a total of 21 people:  11 MCI patients were assigned to the intervention group (mean age 76.4 and mean MMSE score equal to 27.5). 10 healthy people as the control group (mean age 73.4 and mean MMSE score equal to 29.6) | Less than a day | Camera without sensor, IR sensor, pressure sensor |
| 14 | Lazarou/2016/Greece[21] | Developing a system for continuous remote monitoring of difficult daily living activities, and personalized interventions for system feedback and clinical observations, in order to enhance cognitive function and quality of life. | Sensors for sensing presence, object movement, and sleep were used. These technology were implemented in homes to monitor daily activities. | 4 elderly people: 2 with MCI, 1 with mild dementia, and 1 with Alzheimer's disease. | 3 months | RGB camera equipped with sensor, pressure sensor, sensors for vital signs, inertial sensor (accelerometer)  Use of vital sensor to measure EEG |
| 15 | Simoen/2016/Belgium[22] | To alleviate behavioral disorders in persons with dementia using robotics and the IoT. | In a nursing home, a robot was deployed. This robot has the ability to track and detect the activities of patients. | 2 people with dementia living in a nursing home | Unknown | Bluetooth, camera equipped with sensor, IR sensor |
| 16 | Ahmad Akl/2017/ USA[20] | MCI diagnosis with a non-invasive home monitoring system | The person's presence in each of places was recognized by deploying various types of sensors in various locations throughout the home. The information gathered by the sensors was forwarded to a central server. | 68 individuals in total, 15 of whom acquired MCI during the research. The subjects' MMSE score was 24. | 24 weeks | IR sensor, inertial sensor (accelerometer) |
| 17 | Alvarez/2017/Spain[59] | To improve patient quality of life, through ICT4Life infrastructure for data gathering, patient monitoring, and timely notice of activities. | The proposed system was used to detect daily activities and identify the patients.  To diagnose the patient's presence, RGB sensors and a wireless sensor network were used, as well as multiple sensors. To detect patient activity, sensor data was collected. | The number of people who participated in the study is not specified. However, 700 samples were collected. | 24 days | Ordinary camera, camera equipped with RGB-D sensor, switch sensor, sensors for vital signs, inertial sensor (accelerometer),  Using a vital sensor to measure blood pressure, body temperature |
| 18 | Gattinger/  2017/  Switzerland  [72] | Assessing the effectiveness of a movement monitoring system on the sleep quality of nursing home residents with cognitive impairment. | The intervention group's actions were analyzed using the monitoring system, which includes a range of sensors and cameras equipped with sensors. The control group was not given this system.  For each patient in a nursing case conference, professional opinions was exchanged, based on the data collected from the sensors. The patients’ condition, treatment plan, and therapeutic interventions were all deemed suitable for use in the person's care during this meeting. | 44 people with dementia.  The intervention group consisted of 22 people (mean age 86.32 years) while the control group consisted of 22 people (mean age 88.68 years). | 30 days | Pressure sensor, camera equipped with sensor |
| 19 | Lam/2017/  Hong Kong  [60] | Developing an activity monitoring and tracking system (Smart Mind) to assist Alzheimer's patients in living independently in their homes | Motion sensors were used in Smart Mind. The sensors were installed in a tiny suite, and the sensors discovered and reported the person's condition within the room. The system was installed in a small living room with all of the necessary living equipment. To detect the presence of a human in different parts of the room, NFC was employed on particular gadgets such as medicine boxes and Kinect cameras. | A 75-year-old individual (2700 samples of different positions of the person) | Less than a day | NFC, GPS, camera equipped with RGB-D sensor, inertial sensor (accelerometer) |
| 20 | Mendoza/  2017/  Philippines  [61] | Deployment of a wearable tracking system to assist Alzheimer's patients' family members and carers. | Patient tracking through GPS and GSM, as well as sensor data transmission via ZigBee. The patient's physical location is determined using GPS, and data is sent and received using GSM. | 90 Alzheimer's patients. | Unknown | ZigBee, GPS, GSM, inertial sensor (accelerometer |
| 21 | Alberdi/2018/USA[73] | Feasibility study of using smart homes with a variety of sensors to record behavioral data based on a person's situation in order to diagnose and predict Alzheimer's disease early. | Data on patient behaviors is collected and the amount of mobility, cognition, and moods such as depression are measured using the Center for Studies in Adaptive System (CASAS) system, which is a set of various sensors installed in a smart home. | 29 participants | Less than one month to 60 months per person | IR sensor |
| 22 | Alvarez/2018/Spain[62] | To monitor the patients’ physical activity and movements. | A service subsystem, a high-level subsystem, and a low-level subsystem make up a three-tier architecture for this technology. A number of sensors and cameras were used to diagnose and evaluate how a person moves (the volume and quality of movement in the person's complete living space over the course of 24 hours). | 18 patients, both women and men, between the ages of 55 and 94.  The severity of Alzheimer's disease was categorized into three groups (severe, moderate, and primary). | 24 days during 10 weeks | Camera equipped with sensor, IR sensor, switch sensor, inertial sensor (accelerometer) |
| 23 | Amiribesheli/2018/England[23] | Investigating ways to improve the quality of smart homes for dementia patients by applying new layout and computational techniques. | Developing a smart home that considers the needs of people with dementia at all phases of development. The demands of these people, as well as their caregivers, was gathered, analyzed, and incorporated into the smart home design. In two rounds, two scenarios were developed that measure the patient's vital signs and daily activities and should be completed by the patient. The completed house was evaluated and validated using a questionnaire. | The evaluation questionnaire was completed by five geriatricians. | 1 month | GPS, camera equipped with sensor, IR sensor, switch sensor, sensors for vital signs, inertial sensor (accelerometer), Using a vital sensor to measure blood oxygen levels, lung volume, body temperature, blood pressure, blood glucose, heart rate |
| 24 | Nauha/2018/  Finland[48] | Determining the effect of caregivers' use of supportive technologies on the degree of independence and security of patients with memory impairment | In numerous patient care experiments, a range of assistive devices and alarm systems were used. A number of patients at a Memory Help apartment were equipped with a range of monitoring devices. Motion sensors, pressure sensors, alert and reminder producing devices, tablet with web chat functionality, calendar clocks, illustrated albums, ball-weighted blankets, and therapy aprons are all examples of equipment. In addition, there were a range of entertainment cubes. These patients were cared for by eight nurses. Patients were also cared for and monitored at home by family members in another group.  A patient lived alone at home as well, but a team visited him/her to provide care. | five patients in a nursing home.  5 persons (3 males and 2 women) in the Memory Help apartment,  (average 78 years old, with an average MMSE score of 17.8).  four patients who were cared at home(average age of 78 years and an MMSE score of 7.5). | One week for Memory Help Apartment.  one month for the care at home. | Inertial sensor, pressure sensor, GPS, smartphone, illustrated album |
| 25 | Rostil/2018/  England[24] | Providing a system called " Technology integrated health management (TIHN)" to help keep people with dementia safe and comfortable at home. | Data related to environmental monitoring, health, and location of patients is delivered to caregivers over the network as an alarm by installing sensors in the patient's living environment. | 408 persons in all (204 patients with dementia-204 caregivers) | 6 months | GPS, sensors for vital signs  Use of vital sensor to measure blood pressure, body temperature, heart rate, blood oxygen level, weight, sweating rate |
| 26 | Tan/2018/  Singapore[63] | Early diagnosis of MCI in the elderly using non-invasive IoT devices that record continuous data | Multifunction sensors were installed in the home to monitor daily activities in order to detect signs of forgetfulness. | The intervention group consisted of seven adults with mild cognitive impairment; the control group consisted of eleven healthy elderly people. | 2 months | Bluetooth, IR sensor, switch sensor, pressure sensor, sensors for vital signs, inertial sensor (accelerometer), Using a vital sensor to measure heart rate |
| 27 | Varatharajan/2018/  India[64] | Monitoring how a person moves and walks for early diagnosis of Alzheimer's disease. | A pressure sensor was attached to the bottom of people's shoes to monitor the pressure applied to the sensor by their feet while walking. This data was instantaneously monitored by the researchers. | 173 Alzheimer's patients and 150 healthy people. | Less than a day | Pressure sensor |
| 28 | Basharudin/  2019/  Malaysia[75] | Developing an ambient guideline in the kitchen to assist Alzheimer's patients in cooking. | On the kitchen table, a series of sensors and LED were mounted, and participants were asked to do a variety of activities in order. The steps taken by the person were shot with the camera as the tasks were being completed, and the order in which the LEDs were switched on was recorded. To signify that a task was done, the LEDs should be turned on in order from the beginning to the end. | 16 Alzheimer's patients (intervention group)  16 healthy individuals (control group) | Less than a day | Camera equipped with sensor |
| 29 | Enshaeifar/  2019/England[68] | To assess the pattern of daily activities and to diagnose the occurrence of a urinary tract infection using vital sign sensors and an individual activity monitoring system. | Two infrared passive sensors were installed in the hall and living room, four motion sensors in the kitchen, one on the pill box and medicine cabinet, and two on the bedroom and bathroom doors. A sensor was installed on the main entrance door of the patient's residence, as well as in the bed and chair. Participants used Bluetooth-enabled devices to record physiological drugs twice a day. Blood pressure, heart rate, and sweating rate were collected. In the diagnostic model, these physiological data were combined with sensor data. | 28 people with Alzheimer's disease, ranging from mild to moderate. | 30 days | Bluetooth, IR sensor, switch sensor, pressure sensor, sensors for vital signs(Using a vital sensor to measure heart rate, body temperature, weight, sweating rate) |
| 30 | Kaur/2019/  India[50] | Implementation of a an integrated rehabilitation monitoring and Alzheimer's diagnosis system using the cloud-based IoT. | This system (IC-SMART) was structured as a network, allowed stakeholders to communicate with one another. Patient data from sensors, general practitioners' inputs, and AD specialists' inputs were all compiled into a knowledge base and used to develop Bayesian network decision models.  Diagnose, communication, and messaging, as well as the production of real-time and planned messages and routing help were some functions of IC-SMART.  RFID tags were also used to track humans and their motor movements. The cloud subsystem processed data, and analyzed data and performed functions such as detecting the disease, connecting with the patient, creating real-time and planned messages, and assisting with routing. | 374 people with various cognitive disorders  (ages of 60 and 96  with various MMSE scores) | Unknown | RFID, GPS, Bluetooth  IR sensor, pressure sensor, |
| 31 | Kota Aoki/2019/  Japan[74] | To identify the elderly with a low MMSE score using the features extracted from the sensor during Dual Task Gait and Single Task Gait. | Individuals completed a cognitive task while walking and were fitted with whole body motion sensors, from which gait-related variables were collected and evaluated using SVM. The MMSE score and sensor data were used to diagnose Alzheimer's disease. | 103 persons. | Less than a day | Cameras with and without RGB-D sensor |
| 32 | Landero/2019/Spain[49] | Detection of memory defects with a sensor-based IoT system | Thirty items were placed in a sensor-equipped cabinet.  Sensors counted the number of times the cabinet door was opened and closed.  The participants were given a face-name test first, in which they were shown a photo of numerous persons and their names were told to them. The patient was then asked to name the people in the snapshot by being shown the photo.  Following the preceding test, each participant should remove a list of objects from the cabinet and explain to the researcher any questions they asked regarding each item. The length of the test was also recorded. | 23 persons in the study, some of whom had impaired memory. (Average age: 36.17 years old) | Less than a day | Switch sensor |
| 33 | Lazarou/2019/Greece[47] | To evaluate the long-term effects of assistive technology combined with non-pharmacological intervention for persons with cognitive impairments. | The intervention group’s data was recorded and relayed via sensors, lasting 4 to 12 months based on the system's findings.  The control group was not in the smart home, and the therapists received information on their status from the participants themselves using self-report methods.  On the basis of this manual data, a series of routine treatment actions for them was carried out.  There was no mechanism or intervention for the third group. | Six Alzheimer's patients and twelve MCI patients were separated into three groups, one intervention group and two control groups. | Four to 12 months | Cameras with and without RGB-D sensor, switch sensor, sensors for vital signs (Using a vital sensor to measure heart rate) |
| 34 | Sefcik/2020/USA[69] | To identify changes in dementia patients’ heart rates at a nursing home before, after, and during their Persistent Vocalization(PV) | The heart rate was continually measured for 2 hours using the Zephyr Bio Harness system (a light, portable belt with an electrocardiographic sensor.  During the watching period, individual videos were also recorded for 2 hours. These movies were uploaded to the server and compared to heart rate data collected at the time of the PVs. The researcher was able to determine the times of PVs using these films.  The researcher visited with each subject for 9 hours and 45 minutes (with a heart rate of 9-10 in 15 seconds) at the start of the trial to capture routine heart rate patterns as well as PV status. This information was utilized to plan two hours of data collection while wearing a belt containing a sensor and a video recorder. | Two persons aged 65 and up with an MMSE score of less than 20  Has a proven history of PVs | Less than a day(2 hours) | Sensors for vital signs, Record and send video  Using a vital sensor to measure heart rate, Camera |
| 35 | Buchman/  2020/  USA[65] | To predict cognitive health using walking speed and gait and other motor indicators | Data about how the person moves was collected and relayed by attaching a sensor to the individual's belt. | 1249 persons in total, with an average age of 80 years. | 3.6 years | Inertial sensor (accelerometer) |
| 36 | Kroll/2020/  Germany[70] | Providing a non-contact wandering monitoring system.  In the emergency department, a shielding chamber device was used for patients with dementia.  To assess the system's validity and reliability.  Evaluating the system's impact on overall health of the patients, and the patients’ wandering | NCMSys uses a range of sensors to track a patient's vital signs, motions, and verbal stimuli. The researcher receives both sensor and camera data at the same time.  A visual sensor is a camera that records the motions of the patient.  To detect sound stimuli, a sound sensor was put above the patient's bed.  A sensor placed near the patient's mattress that monitors the patient's heart rate, respiration rate, and movement changes without requiring the patient to be connected.  The sensors merely record and communicate differences with the prior state in the case of bodily movements and aural stimuli, not the entire image or sound. | Six healthy people participated to conduct technical validation.  A feasibility study was carried out on 19 people, with a mean age of 77.4 years, including 10 males and 9 women (14 patients with dementia). | Less than a day | Camera equipped with sensor, audio sensor, sensors for vital signs (Using a vital sensor to measure heart rate, respiration rate) |

2D, Two dimensional; AD, Alzheimer disease; ADL, activity of daily living; CASAS, Center for Studies in Adaptive System; COPE, Carers of Older People in Europe questionnaire; EEG, electroencephalogram; GPS, global positioning system; GSM, global mobile service; IADL, instrumental activities of daily living; IoT, internet of things; IR, Infrared; LED, Light Emitting Diodes; MCI, mild cognitive impairment; MMSE, mini mental state exam; MoCA, Montreal cognitive assessment; NFC, near field communication; PC, personal computer; PV, Persistent Vocalization; RFID, radio frequency identification; RGB, red green blue; RGB-D, red green blue-depth; SVM, support vector machine; TIHN, Technology integrated health management

Table S3: Summary of findings on the use of the Internet of Things in Alzheimer's disease

| # | Author/reference | Summary of study | ADL details | Aims of monitoring/ outcome measures | Evaluation results |
| --- | --- | --- | --- | --- | --- |
| 1 | Franco[26] | Various equipment were fitted in a 50m^2^ setting so that the elderly can be maintained and monitored in a safe environment for as long as possible. | Entering and leaving home, studying, watching TV, visiting family and friends, resting, sleeping, personal hygiene, cooking and eating | Alzheimer disease diagnosis(Dementia), Vital signs, Sleep, ADL, Reminder/Alarm, Social interaction | It was feasible to diagnose AD in a person by comparing the amount of activity at different times for early diagnosis of neurological disorders and detection of changes in the body's physiological clock. |
| 2 | Wang[28] | The use of bed sensors to diagnose dementia and the effect of sleep on quality of life and to diagnose sleep patterns (qualitative and quantitative sleep patterns). |  | Alzheimer disease diagnosis(Dementia), Sleep | Sleep monitoring is an excellent criterion for early identification of dementia, according to a study of two people's sleep.  A healthy adult's sleep quality, amount, and rhythm is better to that of AD person. |
| 3 | Taub[27] | Escort, a system with a range of sensors and alerts, was introduced. The patient's carers were interviewed. |  | Agitation, Reminder/Alarm, Tracking | The alarm labeling was classified into six groups after comparing the data obtained with the sensors and the information collected manually:  Good: A good or correct label indicates that the person is in the correct location.  Safe: The person is not in danger or their dangerous situation is resolved as soon as they receive the warning.  Exemption: The warning is correct in terms of location, but it is incorrect regarding the person about whom the warning is issued.  Bad: Wrongly alerts further confuses the caregiver.  Unattended: The caregiver does not check on the person being warned.  Blank: No recorded manual alerts, which are unfortunate on non-accidental days.  The capacity of the patient to follow the Escort system and know which patient was at risky situation as well as the ability to manage the patient's actions were assessed valuable by caregivers. |
| 4 | Sacco[29] | Asking participants to do two ADL scenarios and comparing how they completed them across healthy, MCI, and AD people:  For each case, an evaluation process was explored.  Healthy people and people with AD were compared. Other healthy patients were compared to MCI subjects in the second scenario. | Some of these activities are related to scenario 1 and some are related to scenario two or common to both: studying, making tea, making phone calls, watering pots, watching TV, arranging things, writing shopping lists, preparing medicines | Alzheimer disease diagnosis(MCI, AD), ADL, Movement, Medication | The proposed method in this study can be utilized to detect MCI or AD in the elderly since the DAS score in both protocols was different between healthy and patients with cognitive impairment, and healthy people earned a superior score in executing DAS correctly. |
| 5 | Crispim[53] | People carried out specific tasks and activities. Sensors were used to gather and analyze data relating to the activities. | Watching TV, making a shopping list, writing checks, answering the phone, reading the newspaper, watering the plants, and arranging medicines | Medication, ADL, Movement, Reminder/Alarm | When employing a single sensor or multiple sensors, the F measure was reported.  Identifying daily activities plus sitting  Fmultiple = 71%  Fmono = 51%  Identifying daily activities plus standing:  Fmultiple = 71%  Fmono = 73%  Mean F for daily activity with posture  Fmultiple = 71%  Fmono = 68%  Mean F or daily activity without posture  Fmultiple = 81.2%  Fmono = 88.2% |
| 6 | Robert[54] | A report on the SWEET HOME project.  Various audio and video sensors were employed in this experiment. The level of physical activity of patients is determined. The activities of daily living (both indoors and outdoors) were also assessed.  In these circumstances, the system's accuracy and sensitivity were examined and reported. A feasibility analysis was also carried out. | Making tea, making phone calls, watering plants, using TV controls, arranging items, and leaving the room | ADL, Tracking, Movement | Diagnosis of physical activity performed by the patient with 96.9 to 100% accuracy.  Daily activities with a sensitivity of 90% and an average accuracy of 83.51%.  83% of people considered this technology desirable.  The appropriateness indexes of evaluation and intervention periods and difficulty level were 98 and 96 percent, respectively.  Only for a limited time during the day, 67 percent of participants were willing to be monitored by sensors at their place of residence (not for a long time). |
| 7 | Chan[55] | Using an intelligent system with a range of sensors to collect data on a person's movement.  These people were in a hospital and an Alzheimer's ward, and the environment surrounding them was similar to a smart home. The presence of a person was detected using ZigBee and infrared sensors in various locations. Patients were tracked and their position were determined. There are three degrees of supervision, ranging from high to low:  At the level of high supervision, both ZigBee and (IR) sensors were used.  Only ZigBee data was used at the moderate supervision level.  Only IR sensor data was used at low supervision level. | Individual activity and presence in the bedroom, the bathroom, and on the balcony. Examining the person's walking style. | Sleeping, ADL, Tracking, Movement(gait), Reminder/Alarm | Examining the degree of correlation between various activities:  In the night, there was a 0.71 correlation between getting up and going to the bathroom.  The correlation between waking up in the middle of the night and doing daily activities was -0.35  The correlation between getting up at night and performing the usual activities of the elderly and medical staff was 0.47  Given that collecting some patients’ data is not possible with current care methods such as distance traveled, routes within the care unit, time spent in each area, wandering, and waking up at night, this technology is acceptable by the medical team. |
| 8 | Kaye[56] | The number of days of computer use, and average daily use were evaluated to diagnose MCI. |  | Alzheimer disease diagnosis(MCI) | There was no difference in the number of days of use, average daily use, or changes in the quantity of computer use on different days between the two groups at the start of the study, however over time, MCI participants used the computer fewer than the healthy group.  MCI patients’ average daily computer usage declined with time.  Participants with MCI had more changes in computer usage on different days. |
| 9 | Stucki[57] | Environmental data and data relating to a person's life were collected utilizing 50 sensors inside 10 boxes that were put throughout the home environment. A person's daily activities were divided into eight categories. | Sleeping, making-up, toileting, getting ready for bed, cooking, eating, watching TV, and doing activities while sitting | Sleeping, ADL, Movement, Alzheimer disease diagnosis(Dementia) | System's performance, including sensitivity and specificity was calculated for ADLs. The measures for whole category of monitored activities were as follows:  99.53 % reliability  92.5 % specificity  91.27 % sensitivity  The activity patterns of healthy people were compared to those of people with Alzheimer's disease, and there was a difference between the two groups, with healthy people having distinct activity patterns and those with Alzheimer's disease being difficult to recognize.  For people with Alzheimer's disease, the system was acceptable. |
| 10 | Xin zhu[58] | Umemory is a method that assessed the sleep quality of Alzheimer's patients in nursing homes.  Other actions were also extracted from the data, such as restrooms. Data related to bedtime, stability in bedtime, and sleep efficiency were extracted. The sleep patterns of the patients were also obtained. This system's data was compared to information gathered manually.  A kit with vital signs sensors was placed next to the person's bed to check the individual's general health in addition to sleep monitoring. An accelerometer was used to assess the quality of the person's movement as well as their acceleration. | Sleeping and awakening during the night, as well as going to the bathroom during the night, activities that occur in everyday life, monitoring person's movements | Vital signs, Sleeping, ADL, Movement | The duration in bed was measured, while the patient was steady in bed, and the sleep efficiency was calculated.  The average sleep efficiency was found to be between 32.1 % and 80 %.  In general, healthy people's sleep patterns change less. |
| 11 | Ahmad Akl[66] | Feasibility study of automatic MCI detection in the elderly utilizing a signal processing and machine learning (SVM) approach based on real data from non-invasive sensor technologies at home.  The difference between morning and evening activities (such as leaving the house and in-home activities) as well as walking speed and main activities at home were used to diagnose. |  | Alzheimer disease diagnosis(MCI), Movement(gait) | In general, the AUC was 0.57.  At the best, AUC was 0.81 at week 24.  AUC = 0.97 after selecting important features.  Sensor data combined with clinical data can help diagnose MCI. |
| 12 | KaraKostas[67] | Before and after various domestic duties, cognitive tests were conducted. | Kitchen works, watching TV, ironing, vacuum, radio, bathroom, bed, boiler, washing clothes, opening and closing doors are all examples of activities. | Sleeping, Medication, ADL, Movement, Social interaction | The evaluations showed improvements including: 1- The better patient sleep through the night; 2- There were fewer times when the patient wakes up throughout the night. 3- Improved daily tasks such as ironing, cooking, and washing machine 4- Watching less television 5- Increasing personal well-being 6- Reducing the symptoms of depression and loneliness 7- Participating in various programs and increasing social interactions with others 8- More effective handling of personal issues and problems. |
| 13 | Jekel[71] | People in both the intervention and control groups were instructed to complete six activities in a smart home equipped with a variety of sensors. The differences between the healthy and patient groups were investigated by collecting and reviewing the data produced from this monitoring.  The feasibility was determined using a questionnaire. | Arranging objects, making coffee, calling people, making a sandwich, watching the TV and using the TV controls, and looking for things. | ADL | There was a difference in how healthy people and MCIs performed these ADLs.  MCI patients required significantly more time (2005 vs. 938 seconds) and had a lower score (48 vs. 58).  Differences between groups were also discovered in the examination of micro-activities.  The IADL questionnaire and cognitive status were used to determine the effectiveness of the activities. The feasibility study indicated that:  Not feeling at comfort while participating in activities (M = 1.2 SD = 0.5) and Long-term scenario (M = 1.1, SD = 0.3, out of 5).  Retrieval activities were rated difficult by 52.4 percent of respondents.  Making a phone call was challenging for 14.3% of respondents. |
| 14 | Lazarou[21] | The data from sensors was used to diagnose sleep patterns, physical activity, and daily activities.  Psychological interventions were used to improve a person's quality of life, cognitive function, and daily functions by integrating these findings with clinical observations. Improvements in cognitive and neuropsychological functioning were also assessed. | Taking medication, going to the kitchen, bathing, watching television, and making tea | Vital signs, Sleeping, Medication, ADL, Tracking, Movement, Reminder/Alarm, Fall, Social interaction, Agitation | From the start of the monitoring through the end of the trial period, the individual's neuropsychological performance improved. This progress was particularly noticeable in the field of physical abilities. Using the system to identify unusual cases and using the data to perform the appropriate treatment measures resulted in improved cognitive status and issues such as the patient's sleep quality. |
| 15 | Simoen[22] | The patient was cared for and monitored using a robot that could move around in different portions of the patient's environment. The work of this robot was recorded in two cases. | Recognizing a person's presence in several locations,  keeping track of individual activities,  Monitoring individual activities in virtual worlds | Sleeping, ADL, Movement, reminder/alarm, Agitation | The robot was capable of completing the following tasks: Returning the wandering patient back to his or her bed, recognizing and playing the elderly's favorite music at their preferred times. |
| 16 | Ahmad Akl[20] | Monitoring and comparing the ADLs of a healthy person with AD patients and classifying the activities based on the distribution of the person's presence in different parts of the home, and comparing the results with tests for the diagnosis of MCI | The person's presence in the bathroom, bedroom, kitchen, and living room, as well as their activity within each of these spaces | Alzheimer disease diagnosis(MCI), Sleeping, Medication, ADL, Movement, Reminder/Alarm, Apathy, Agitation(aggression) | The person's behaviors were computed based on the duration of the patient's presence in various parts of the house, and the person's cognitive status was diagnosed based on this presence. The mean ROC curve and the precision-recall curve for the proposed system for the diagnosis of mild cognitive impairment were 0.716 and 0.706, respectively. |
| 17 | Alvarez[59] | The ICT4LIFE project employs a variety of sensors to provide automatic and intelligent remote monitoring, as well as the potential for early identification and prevention of AD outcomes. To detect abnormal behaviors, ICT4LIFE was employed. From vital signs to patient locations at home were evaluated.  For classification, logistic regression was utilized. | The presence of the patient at home in various scenarios is used to investigate normal and abnormal behaviors. | Vital signs, ADL, Tracking, Fall, Reminder/Alarm, Apathy | The accuracy of diagnosis between normal and abnormal behaviors was 98.4%, while the precision of diagnosis between normal and abnormal behaviors was 98.7 % and recall was 98.3%. |
| 18 | Gattinger[72] | A sensor and a camera were used to keep track of the person’s sleep. The length of time spent in bed, the maximum amount of time spent sleeping without moving, getting out of bed, the number of times a sleeping position was changed in an hour, the percentage of minutes spent moving, the percentage of minutes spent moving with high movement, and the percentage of minutes spent moving with low movement were all investigated. As a secondary outcome, the risk of falls and ulcer pressure were assessed.  The monitoring system was taught to nurses. Before the intervention, preliminary data were collected from the intervention group. | Sleeping and waking hours, sleepiness during the day. | Sleeping, ADL, Fall, Movement | The impact of the system on sleep quality did not differ significantly between the intervention and control groups (p = 0.824), and the control group generally maintained stability during the intervention.  The probability of falling was reduced in the intervention group (p = 0.034).  In the cases of nighttime sleeplessness and ulcer pressure, there was no significant difference between the intervention and control groups. |
| 19 | Lam[60] | The Smart Mind system can detect a person's position while they are sitting, sleeping, walking, standing, or falling.  Using machine learning techniques this system was used to determine a person's location at home. | Eating, showering, sitting and getting up, sleeping, and watching TV | Sleeping, Medication, ADL, Tracking, Movement(gait), Fall, Reminder/Alarm | The status and location of the participants were assessed. The average accuracy of detection of sitting, sleeping, or standing positions was 97 percent with the NB algorithm and 99.1 percent with the SVM. |
| 20 | Mendoza[61] | On the map, a safe zone for 90 patients was marked. Patients who were out of range were detected by a device connected to their belt, and their location was determined and their caretakers were contacted via a cellular network. |  | ADL, Tracking, Agitation | The system detected 86 patients who get out of range, while four patients were not detected.  There was a significant difference between patients and those detected outside the safe range by the system.  The cross validation evaluation showed 90% accuracy for detection patient outside of the range.  An IoT-based monitoring system for the detection of patient's presence yielded positive results, as follows:  The average detection time of patient's departure from the safe region was 1.97 sec, which was enough to make a notification.  In 82% of the cases, the presence of a patient could be detected at the distance of 48-55 m. |
| 21 | Alberdi[73] | For six months, the persons were monitored in a smart home, and the data obtained from the sensors were used to predict the symptoms and development of Alzheimer's disease (with regression model). | The following items were monitored:  • Daily routine:  The complication of daily activities per day, the total number of activities and the number of non-repetitive activities  Maximum and minimum time spent doing nothing during the day, and comparing with the prior day  • Mobility: the total number of activated sensors and the total distance traveled within the apartment.  • Being outside during the day: time spent outside during the day  • Mobility and being outside: Time spent outside the home during the day  • Mobility and being outdoors  Sleeping: The duration and number of daily sleeps  • Overnight toileting: Time spent in nighttime toileting activities.  • Overnight patterns (includes overnight sleep and rest).  Cooking and eating | Alzheimer disease diagnosis(AD), Sleeping, ADL, Memory, Movement | The findings of the study revealed that smart home data can predict movement, cognitive, and depression symptoms. Changes in a person's motor posture and memory skills can be seen in this data.  Not all activities are equally useful in predicting Alzheimer's symptoms, according to the findings.  Movement data, on the other hand, had a stronger impact on predicting Alzheimer's symptoms.  MCI can be diagnosed by IoT-based machine learning techniques; e.g., in a study, random forest (RF) showed the best performance (precision-recall curve=0.73, F-score=0.77, sensitivity=0.92) for this purpose. |
| 22 | Alvarez[62] | Using a variety of sensors and cameras, authors detected unusual actions in persons.  The study was closely observed day and night movement, wandering patient, apathy symptoms, frequency of bathroom visits, leaving house, falling, difficulty in walking, imbalance, involuntary acceleration, activity recognition. The accuracy of each of these behaviors (both normal and abnormal) was evaluated. | The activities of the person in the bathroom, kitchen, bedroom, and living room. | Sleeping, ADL, Tracking, Movement(gait), Fall, Apathy, Agitation | Accuracy (detection of normal and abnormal behaviors) =98.4%  Precision= 98.7%  Recall=98.3% |
| 23 | Amiribesheli[23] | The followings were monitored:  Repeating a phrase  Getting dehydrated  Communication and familiarity  Losing personal belongings  Discovering new ways to interact  Remembering time and date  Learning new experiences  Experiencing night-time wandering events  Forgetting names  Monitoring vital signs including oxygen saturation, body temperature, and blood pressure.  The individual's sleep pattern and general health symptoms were also monitored. |  | Sleeping, Vital signs, Tracking | In each of the two rounds, the difficulty of implementing the system and its effectiveness were assessed. The average score for the system's difficulty was nearly half of the total score, and the average score for the system's effectiveness was 15 out of twenty. |
| 24 | Nauha[48] | The Carers of Older People in Europe questionnaire(COPE) assessment was used to assess the influence of using assistive technologies on the burden of patient care.  Caregivers answered this questionnaire twice, once at the start and once at the end of the trial.  The benefits of IoT based assistive devices were assessed using questionnaires, diaries, and recorded data.  The following were the topics covered in this research:  • The impact of utilizing assistive technologies on medical staff  • The impact of using assistive technologies on caregivers at home and in the community  The impact of adopting assistive technologies in assisting people with memory impairment in protecting their independence and security | Doing cleaning, planning, according to warnings and reminders,  sleeping, having fun | ADL, Tracking, Fall, Reminder/Alarm, Sleeping, Memory, Movement | In the instance of GPS, which was used to locate patients, it was only used by patients who lived at home, and it was provided with two-way communication by linking it to internet maps and establishing an alert button for usage in emergencies.  With the use of bracelets, two people with dementia were able to walk independently daily.  The initial difficulties that limit the adoption of these gadgets were technical installation issues, complex user interfaces, and poor sound quality alerts.  Two patients were found as being capable to use GPS for establishing patient safety and security as well as conducting tasks independently.  Three of the three caregivers thought GPS was simple to use. |
| 25 | Rostil[24] | In the TIHN project for dementia care, patient-related factors were explored using a variety of sensors, ranging from vital sign monitoring to outdoor positioning. | The person's movements were tracked indoors and outdoors. | Vital signs, ADL, Tracking, Fall, Reminder/Alarm, Agitation(aggression) | The alarm signals that were received were categorized.  Clinical concerns were the source of 61% of the warnings. Technical cases accounted for 26%, while environmental matters accounted for 14%. Blood pressure and blood oxygen levels were mentioned in 14% of the warnings. |
| 26 | Tan[63] | Motion, door, medication box, bed occupation, key tracker, heart rate, pedometer, and frequently missing personal items when leaving the house, as well as forgetting to take medicine were compared between the elderly with and without MCI. | Patient movement in the living room, kitchen, and bathroom, as well as sleeping, taking medicine, and social interactions | Alzheimer disease diagnosis(MCI), Vital signs, Sleeping, Medication, ADL, Movement, Memory, Social interaction | When the actions of persons with MCI were compared to the control group, it was discovered that those with MCI had the highest rate of forgetfulness of personal things.  Furthermore, it was discovered that those with cognitive impairment had the highest rate of medication forgetfulness. |
| 27 | Varatharajan[64] | This project collected data on how healthy people and Alzheimer's patients move and walk, compared the data, and classified people into two healthy and Alzheimer's groups, using the DTW algorithm. |  | Alzheimer disease diagnosis, Movement(gait) | The findings of the DTW algorithm were 94 for specificity and 95.9 for sensitivity. This algorithm outperformed than other algorithms |
| 28 | Basharudin[75] | Two groups of healthy and Alzheimer's patients were compared in terms of moving objects in the kitchen. | Cooking | ADL | In patients with Alzheimer's disease, the number of completed activities was much lower. The number of completed tasks did not differ significantly between men and women with Alzheimer's disease. |
| 29 | Enshaeifar[68] | Urinary tract infection was detected using data collected from sensors, everyday activities, and a machine learning model on the sleep-related data. | The amount of actions in the living room, kitchen, and bathroom. | Vital signs, Sleeping, Medication, ADL, Fall, UTI | Comparing the sleep analysis obtained from automatic data with the individual's self-reports indicated  Sensitivity = 0.73, specificity = 0.88, accuracy = 0.85 |
| 30 | Kaur[50] | The software service was developed for an Android-base smart phone and put on the Amazon cloud to investigate the feasibility of the proposed solution. There were two subsystems: An IoT subsystem and a cloud subsystem.  Between the two subsystems, an interactive human-computer interface was developed. This interface received the information from the IoT subsystem and transmitted it to the cloud for processing and storage.  For qualitative evaluation, 100 participants were interviewed and usability, accuracy, readability, and convenience were evaluated as excellent, good, or poor. | Sleeping and behavioral moods | Alzheimer disease diagnosis(AD), ADL, Sleeping, Tracking(patient presence detection, routing), Reminder/Alarm, Movement | The overall F-measure was 0.934.  Individuals were grouped into four categories based on whether they lived alone or with family members, and whether they did or did not activities.  People who lived alone and undertook activities had the highest score for usability (42%).  People who lived with their families and participated in activities had the highest accuracy (34%).  People who lived with their families and participated in activities had the highest readability (55%).  People who lived alone and were active had the highest convenience score (40%). |
| 31 | Kota Aoki[74] | Data on persons’ gait was extracted, and the subjects were instructed to execute a cognitive activity in addition to walking in dual task mode. In single task mode, participants were asked to walk without additional task. |  | Alzheimer disease diagnosis(AD), Movement(gait) | For walking, AUC and ROC was reported.  For people with a greater MMSE score equal to 25, an AUC of 0.747 was found for Alzheimer's diagnosis in dual task.  For the single task, an AUC of 0.598 was achieved. |
| 32 | Landero[49] | Installing a sensor on a cupboard door and analyzing the data to test persons’ memory and comparing the results to the Face-Name test.  Thirty different things were placed in the cupboard, and the participants were instructed to take each one out and explain how it worked and was used.  The correlation between smart cupboard test and self-reported test, as well as the correlation between system accuracy and Face-Name test was calculated. | Looking and searching items inside of smart cupboard | Alzheimer disease diagnosis(AD), ADL, Memory | Faces name test and sensor intervention had a correlation of 0.597, whereas response time and Face-Name test had a correlation of 0.341. |
| 33 | Lazarou[47] | Daily activities were tracked as part of a complete smart home system developed to assist people with AD and MCI.  This system performs a variety of supportive actions to assist patients in getting a better night's sleep.  A number of clinical interventions were carried out based on the information gathered from the patients’ condition. | Cooking, watering plants, taking medicine, sleeping, daily physical activity, personal activities, and watching television | Sleeping, Medication, ADL, Movement, Reminder/Alarm, Memory, Vital sign | Neuropsychological tests in the intervention group showed significant changes before and after the intervention, and these individuals had a better cognitive state after the intervention.  The intervention group's psychological test results were much better than the other two groups at the end of the study.  Individuals' sleep patterns improved significantly following the intervention.  The correlation between light sleep length and cognitive tests was 0.836, while the correlation between total sleep duration and cognitive tests was 0.843, according to sleep monitoring by an IoT system. |
| 34 | Sefcik[69] | Participants’ data was gathered for at least 2 hours on days without PVs and compared to their data obtained for at least 2 hours on days with PVs.  Two people reviewed and analyzed 2 hours of heart rate data as well as related movies of the patients. The individual's heart rate was measured in three different time periods: before, after, and during the PV event. |  | Agitation(Persistent vocalizations), Vital sign | At times of PV, the heart rate was around 40 beats per minute (41 times for the first participant and 39 times for the second). In addition, on days when there was no PV, both subjects had regular sleep and quiet waking.  Heart rate data can be evaluated with this systems, and PV can be detected. |
| 35 | Buchman[65] | Participants were asked to do 12 specified activities, including walking, sitting, and standing in various positions. A sensor was used to collect data from motion tests. In a before and after design, psychological tests were compared. | Sitting, standing up, and walking tasks | Alzheimer disease diagnosis(AD, MCI), Movement(gait), ADL | In a univariate analysis, 9 out of 12 movements were considered useful in predicting MCI and Alzheimer's disease.  In terms of mobility, five of the twelve movements were found to be beneficial in predicting MCI and Alzheimer's disease.  Over the course of 3.2 years, 25% of persons acquired MCI, and two of the 12 movements were useful in predicting MCI in these people.  9.4% of the participants had demented, and four out of the twelve movements were useful for predicting dementia.  In multivariate analysis, 6 out of 12 movements had a significant relationship with ADL impairment. In terms of mobility, 5 of the 12 cases correctly predicted MCI and Alzheimer's disease.  Two of the twelve movements were beneficial in predicting the disease of people with mild cognitive impairment.  Two of the 12 movements were beneficial in predicting the disease of these persons with dementia.  Overall, the findings of this study demonstrated that using sensor technology and gait analysis, mobility issues and difficulty in ADL can be used for the earlier detection of MCI and Alzheimer's/dementia. |
| 36 | Kroll[70] | Vital signs, body movements and voice of patients with dementia, as symptoms of wandering, were observed in an emergency department.  The NCMSys system and a protection chamber in the patient’ bed for separating dementia patients from emergency congestion. |  | Alzheimer disease diagnosis(AD, MCI), Movement(gait), Vital sign, Agitation | The reliability assessment took two hours and was completed in one day. The patient was monitored by the NCMSys system for two hours, one hour without ChD and the second hour with ChD.  The findings of the reliability assessment revealed that NCMSys was capable of measuring heart rate, changes in movement, and audio stimuli (R2 was 0.874 without ChD and 0.608 with ChD).  For measuring respiration rate, R2 was 0.840 in the absence of ChD and 0.602 in the presence of ChD. |

AD, Alzheimer disease; IADL, activity of daily living; AUC, area under the ROC curve; CASAS, Center for Studies in Adaptive System; ChD, charite dome; COPE, Carers of Older People in Europe questionnaire; DAS, daily activity scenario; DTW, dynamic time warping; GPS, global positioning system; IADL, instrumental activities of daily living; IoT, internet of things; IR, infrared; MCI, mild cognitive impairment; MMSE, mini mental state exam; NB, naïve bayes; NCMSys, non-contact monitoring system; NFC, near field communication; PV, Persistent Vocalization; RFID, radio frequency identification; ROC, receiver operating characteristic; SVM, support vector machine; TIHN, Technology integrated health management; UTI, Urinary tract infection
